# Modeling Oncogenic Signaling in Colon Tumors by Multidirectional Analyses of Microarray Data Directed for Maximization of Analytical Reliability

**DOI:** 10.1371/journal.pone.0013091

**Published:** 2010-10-01

**Authors:** Magdalena Skrzypczak, Krzysztof Goryca, Tymon Rubel, Agnieszka Paziewska, Michal Mikula, Dorota Jarosz, Jacek Pachlewski, Janusz Oledzki, Jerzy Ostrowsk

**Affiliations:** 1 Department of Gastroenterology and Hepatology, Medical Center for Postgraduate Education, Warsaw, Poland; 2 Laboratory of Bioinformatics and Systems Biology, Maria Sklodowska-Curie Memorial Cancer Center and Institute of Oncology, Warsaw, Poland; 3 Department of Oncological Genetics, Maria Sklodowska-Curie Memorial Cancer Center and Institute of Oncology, Warsaw, Poland; 4 Department of Colorectal Cancer, Maria Sklodowska-Curie Memorial Cancer Center and Institute of Oncology, Warsaw, Poland; Memorial Sloan-Kettering Cancer Center, United States of America

## Abstract

**Background:**

Clinical progression of colorectal cancers (CRC) may occur in parallel with distinctive signaling alterations. We designed multidirectional analyses integrating microarray-based data with biostatistics and bioinformatics to elucidate the signaling and metabolic alterations underlying CRC development in the adenoma-carcinoma sequence.

**Methodology/Principal Findings:**

Studies were performed on normal mucosa, adenoma, and carcinoma samples obtained during surgery or colonoscopy. Collections of cryostat sections prepared from the tissue samples were evaluated by a pathologist to control the relative cell type content. The measurements were done using Affymetrix GeneChip HG-U133plus2, and probe set data was generated using two normalization algorithms: MAS5.0 and GCRMA with least-variant set (LVS). The data was evaluated using pair-wise comparisons and data decomposition into singular value decomposition (SVD) modes. The method selected for the functional analysis used the Kolmogorov-Smirnov test. Expressional profiles obtained in 105 samples of whole tissue sections were used to establish oncogenic signaling alterations in progression of CRC, while those representing 40 microdissected specimens were used to select differences in KEGG pathways between epithelium and mucosa. Based on a consensus of the results obtained by two normalization algorithms, and two probe set sorting criteria, we identified 14 and 17 KEGG signaling and metabolic pathways that are significantly altered between normal and tumor samples and between benign and malignant tumors, respectively. Several of them were also selected from the raw microarray data of 2 recently published studies (GSE4183 and GSE8671).

**Conclusion/Significance:**

Although the proposed strategy is computationally complex and labor–intensive, it may reduce the number of false results.

## Introduction

Colorectal cancer (CRC) arises as a multi-step process of successive cellular clone selection. As a result of the growth advantage of dysplastic cells over their normal neighbors, the morphological counterpart of molecular alterations leads to progressive cytological and architectural derangement recognizable as the adenoma-carcinoma sequence [1], [2]. Recently, no more than a dozen or so somatic “driver” mutations were established as being responsible for CRC development [3], [4]. However, tumors exhibiting homogenous phenotypes share few mutated “cancer genes”; therefore, cancer complexity at the gene level is likely reduced to a limited number of alterations within signaling and metabolic pathways [5].

An individual cancer phenotype is the result of cell-specific, developmental stage-specific, and metabolism-related changes in gene expression selectively occurring at a time and modified by epigenetic interactions [6]. With the introduction of high-density DNA microarrays, an expectation of insight into the overall molecular components of carcinogenesis has developed. Unfortunately, a comparative analysis of microarray-based studies on CRC development found rather weak overlap of the gene expression profiles ([7] and the results section). These discrepancies in the identified expression profiles may be due to technical reasons, including the use of various microarray platforms, different tissue collection methods, and numerous analytical algorithms [7]. Although the rate of false assumptions might be minimized by using an “optimal” analytical protocol, the selection of such protocol is still challenging [6]. Consequently, microarray experiments allow for rough and mostly indirect assumptions. Therefore, one may ask whether multidimensional and sub-optimal microarray-based data can be applied to the study of complex biological systems, including carcinogenesis.

To answer this question, microarray data originating from two experimental procedures were analyzed by multiple methods employed for identification of consensus differences in pathways underlying CRC development through the adenoma-carcinoma sequence. Samples of normal mucosa, adenomas, and carcinomas obtained during surgery or colonoscopy were processed to select differences between: (i) epithelium and mucosa in normal tissue away from and directly adjacent to carcinoma, in adenoma and carcinoma (using microdissected samples) and (ii) normal and neoplastic tissues and adenomas and carcinomas (using whole tissue sections). Finally, we addressed the potential and challenges of translating microarray-based gene expression profiles into the functional aspects of carcinogenesis.

## Results

A total of 170 GeneChips were hybridized in this study. Twenty-five of 130 arrays representing whole tissue section samples were rejected from the data analyses. Twenty-four of these arrays were rejected on the basis of poor GeneChip quality according to the parameters established by Affymetrix and due to their internal inconsistency with others as established by the principal component analysis (PCA) (not shown). One microarray was rejected because of mechanical damage. A parameter summary of 145 arrays of suitable quality, 40 and 105 represented microdissected and whole tissue section samples, respectively, is provided in Table S1.

From 54,675 probe sets of the Affymetrix HGU133plus2 microarray, 31,962 and 25,410 probe sets for whole tissue sections and 29,242 and 24,002 probe sets for microdissected samples passed the filtering procedure according to MAS5.0 and GCRMA+ LVS algorithms, respectively. As shown in Figure S1, the probe set signal distribution and levels extracted with the two normalization algorithms significantly differed.

### Signaling pathways distinguishing between colonic epithelial cells and mucosa

Probe set selection from microarray data representing tissue samples may be significantly affected by differences in the cell type content of the normal and dysplastic mucosa (Table 1). Bearing that in mind, we intended to define the consensus differences in KEGG pathways which are more conserved between colonic epithelial cells and mucosa than between normal and neoplastic tissues. Gene expression profiles corresponding to tissue morphology were established in pure colonic crypt epithelial cells (CEC) and mucosa (MUC) (representing the epithelial cell layer and cell content in lamina propria) which were captured from various parts of the tumor consisting of invasive adenocarcinoma adjacent to tubular adenoma with low grade dysplasia and from paired full-thickness normal colon using the laser capture microdissection.

**Table 1:** The relative cell type content within normal and dysplastic mucosa used for laser capture microdissection (per 1 mm^2^ of the area).

Cell type	Tissue sample type			
	Normal colon	Normal colon dissected from tumor	Adenoma	Carcinoma
Epithelial cells (percent of total)	3183 (71.4%)	3032 (63.3%)	1650 (53.4%)	2330 (81.4%)
Fibroblasts	240	880	280	133
Lymphocytes	266	420	765	164
Intraepithelial lymphocytes	80	60	24	31
Plasmocytes	594	173	133	92
Granulocytes	20	26	37	35
Histocytes	47	40	78	45
Endothelial cells	26	160	120	33
Total	4456	4791	3087	2863

Data sets from these microdissected samples were analyzed in the following pairs: CEC *vs*. MUC dissected from distant full-thickness normal colon (NC), normal colon mucosa dissected from tumor (NT), adenoma (AD), and carcinoma (CA). Pair-wise comparisons were performed separately on microarray data sets normalized by MAS5.0 and GCRMA+LVS algorithms. Probe sets sorted according to significance of differentiation of CEC and MUC in any pair set were used in Kolmogorov-Smirnov (K-S) test to evaluate KEGG pathways alterations. The results of KEGG annotations for each comparison are summarized in Table S2. The pathways found in at least three of the four pair-wise comparisons (Table 2) were considered to be distinguishing between colonic epithelial cells and mucosa and were excluded from the further selection of oncogenic signaling pathways.

**Table 2:** KEGG pathways found significant (KS test) in pair-wise comparisons of pure colonic crypt epithelial cells (CEC) and mucosa (MUC) in at least three of four sample groups: normal colon (NC), normal tumor mucosa (NT), adenoma (AD), and carcinoma (CA).

KEGG term	NC	NT	AD	CA
Cell adhesion molecules (CAMs)	+	+	+	+
ECM-receptor interaction	+	+	+	+
Focal adhesion	+	+	+	+
Allograft rejection	+	+	+	+
Autoimmune thyroid disease	+	+	+	+
Complement and coagulation cascades	+	+	+	+
Asthma	+	+	+	+
Graft-versus-host disease	+	+	+	+
Type I diabetes mellitus	+	+	+	+
Leukocyte transendothelial migration	+	+	+	+
Hematopoietic cell lineage	+	+	+	+
Systemic lupus erythematosus	+	+	+	+
Cytokine-cytokine receptor interaction	+	+	+	−
Neuroactive ligand-receptor interaction	+	+	+	−
Oxidative phosphorylation	+	+	−	+
Pathogenic *Escherichia coli* infection – EHEC	+	+	−	+
Antigen processing and presentation	+	+	−	+

### Oncogenic signaling pathways

**Figure 1:**
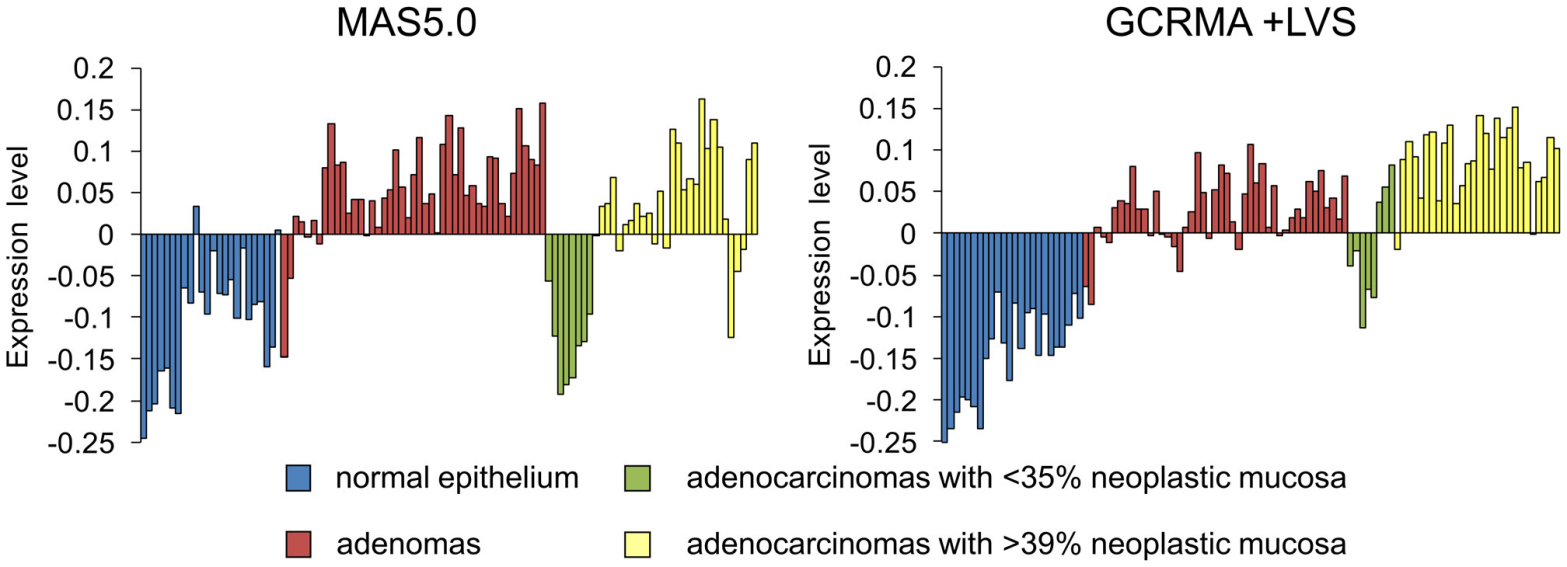
Diagrams of the first statistically independent variability modes extracted from the original normalized with MAS5.0 (left panels) and GCRMA+LVS (right panels) algorithms using SVD, uncovering the microarray data of the whole tissue section samples.

To establish oncogenic signaling distinguishing between normal colon and neoplasms, data sets representing whole tissue sections of normal colon, adenomas, and adenocarcinomas and normalized by both MAS5.0 and GCRMA+LVS algorithms were decomposed into major statistically independent variability modes (supergenes) using singular value decomposition (SVD). We assumed that SVD may establish some co-variations in gene expression that could enable better definition of expression-based functional alterations [8]–[10]. A graphic summary (Figure 1) of the relationships between the samples revealed that the first and strongest mode distinguished between normal colon and neoplastic tissues, with one exception; tumors with lower malignant tissue content (<35%) were grouped mainly with samples of normal colon. This group of samples was discarded from the further pair-wise comparisons. Lists of the probe sets sorted by both SVD and gene-by-gene statistical testing are given in Table S3.

**Figure 2:**
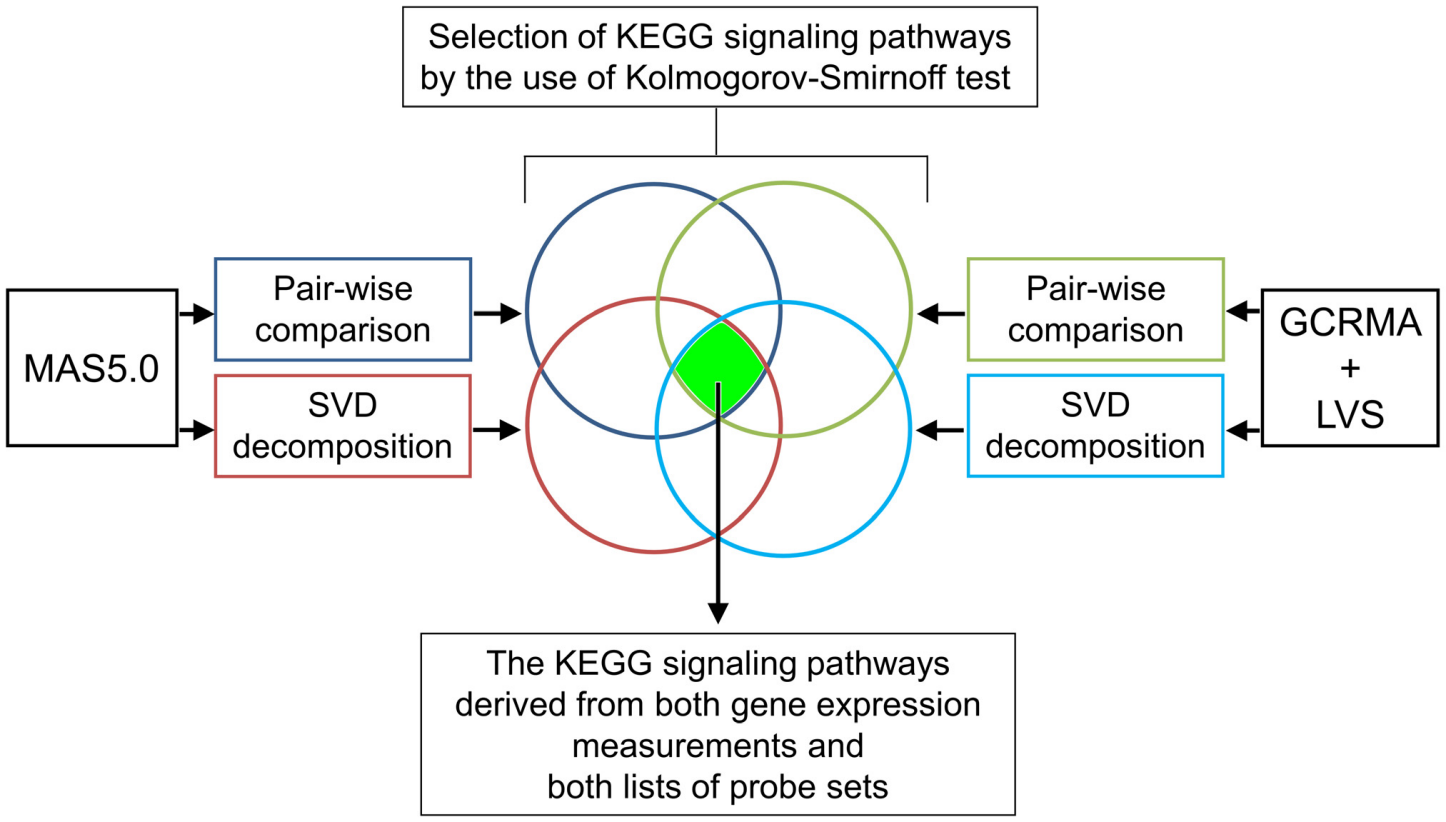
Flow chart of the steps applied to identify oncogenic signaling distinguishing between normal colon and neoplasms. To select the alternated KEGG signaling pathways, two probe-level processing procedures (MAS5.0 and GCRMA+LVS) in connection with two probe set selection and sorting criterions (pair-wise comparison and SVD decomposition) were utilized. Selection of the KEGG signaling pathways was done using the one-sided Kolmogorov-Smirnov test. To obtain the final results, KEGG intersection distinguishing between colonic epithelial cells and mucosa (Table 2) was subtracted from intersection of KEGG annotation results for whole tissue section settings computed for the both comparisons.

The probe sets selected by the first mode of SVD and those differentially expressed between normal and neoplastic tissues were used for the calculation of their attribution to pre-defined KEGG signaling pathways using the KS test, as summarized in Figure 2. The distance of distribution was considered most significant if the corrected *p*-value was less than 0.01 in at least one data set normalized by either the MAS5.0 or GCRMA+LVS algorithm, or if it was less than 0.05 in both data sets. The results of selected KEGG annotations are shown in Table S4. The KEGG signaling pathways derived from both gene expression measurements and both lists of probe sets (selected by SVD and pair-wise comparisons) seem to represent the most reliable findings on molecular alterations between normal and neoplastic colon tissue (Table 3).

**Table 3:** KEGG terms corresponding to genes differentially expressed between normal colon samples with colonic neoplasms and between adenomas and carcinomas.

Normal colon *vs.* colonic neoplasms	Adenomas *vs.* adenocarcinomas
p53 signaling pathway	p53 signaling pathway
Biosynthesis of unsaturated fatty acids	Biosynthesis of unsaturated fatty acids
Proteasome	Proteasome
Cell cycle	Adherens junction
DNA replication	TGF-beta signaling pathway
Purine metabolism	PPAR signaling pathway
Pyrimidine metabolism	Wnt signaling pathway
RNA polymerase	Calcium signaling pathway
Aminoacyl-tRNA biosynthesis	Colorectal cancer
Nucleotide excision repair	Pancreatic cancer
Mismatch repair	Bladder cancer
Base excision repair	Valine, leucine, and isoleucine degradation
Homologous recombination	Fatty acid metabolism
Folate biosynthesis	Tryptophan metabolism
	Sphingolipid metabolism
	Arachidonic acid metabolism
	Axon guidance

Next, to estimate molecular alterations underlying tumor progression to malignancy, data sets representing whole tissue sections from colonic neoplasms were decomposed into SVD modes (Figure S2) and analyzed by pair-wise comparisons (Table S5). Probe sets sorted according to the first SVD mode and those differentially expressed between adenomas and carcinomas were functionally analyzed by annotation to the KEGG signaling pathway database (Table S6). Again, the KEGG pathways were assumed to be the most discriminative between benign and malignant colon tumors if they were derived from the lists of probe sets selected by both SVD and pair-wise comparison (Table 3). In general, they represented signaling networks and cellular metabolism.

### Testing oncogenic signaling in the colon adenoma-carcinoma sequence by the use of the published microarray data

Poor reproduction of microarrays by quantitative RT-PCR was observed in this (not shown) and previously in independent studies [11]–[13], likely as a consequence of the quite different methodology used by these two techniques. Moreover, lack of proper control analytical methods which would be considered as a “gold standard” for functional analysis of microarray data unable direct justification our assumption that the consensus pathways identified by multiple analyses are more likely discriminative than those identified by individual methods. Therefore, we asked a question whether the consensus pathways can be also derived using the proposed strategy from the readouts of the coherent studies.

We conducted normalization, summation and filtration of the raw datasets provided by four recently published microarray-based studies [14] (GSE8671, Sabates-Bellver et al.), [15] (GSE4183, Galamb et al. 2008-1), [16] (GSE15960, Galamb et al. 2010), [17] (GSE10714, Galamb et al. 2008-2), according to our processing algorithm, independently of procedures applied by the respective authors. Expectedly, although all four studies have analyzed colon tumor transcriptomes using the same Affymetrix HGU133plus2 platform, there are less than 1/10 probe sets simultaneously selected significant for all comparisons (Figure S3). Such a little data reproducibility is a well known problem when tissue samples are either prepared or processed in different laboratories or under various conditions.

Next, to test the potential of the proposed analytical procedure in identification of consensus signaling alterations, we employed the microarray data provided by Galamb et al. (GSE4183) [15] and Sabates-Bellver et al. (GSE8671) [14]. Samples in those studies were handled in similar way as in our experiment with the whole tissue sections. As shown in Table 4 (and Table S7), the multidirectional computations selected 11 out of 14 consensus KEGG pathways, which distinguished normal colon and neoplasms in our data sets, also from the GSE4183 data set. Similar KEGG pathways selection was obtained by multiple analyses performed on the GSE8671 data set which established transcriptomes of normal colon and adenomas. This time, 3 of 4 procedures used for data normalization and probe set sorting allowed identifying 11 consensus signaling pathways consistent with those selected in our studies (Table 4), although none common signaling pathways were found when the GSE8671 data set was normalized with the GCRMA+LVS algorithm and probe sets were sorted by the statistic of pair-wise comparison.

**Table 4:** Comparison of KEGG pathways distinguishing between normal colon samples and colonic neoplasms selected from our and two published microarray data.

This study	GSE4183 NC *vs.* AD+CA	GSE8671 NC *vs.* AD
Cell cycle	**+**	**+**
DNA replication	**+**	**+**
Purine metabolism	**+**	**+**
Pyrimidine metabolism	**+**	**+**
RNA polymerase	**+**	**+**
p53 signaling pathway	+/−*	+
Proteasome	+	+
Aminoacyl-tRNA biosynthesis	+	+
Mismatch repair	+	+
Nucleotide excision repair	+	+
Base excision repair	+	+/−*
Homologous recombination	+	-
Folate biosynthesis	-	+
Biosynthesis of unsaturated fatty acids	-	-

*KEGG terms selected only from data sets normalized with MAS5.0.

KEGG signaling pathways related to cell cycle, DNA replication, purine metabolism, pyrimidine metabolism, RNA polymerase, proteasome, aminoacyl-tRNA biosynthesis, mismatch repair and nucleotide excision repair were selected in both our and two recently published microarray-based studies.

In contrast to nearly full agreement described above, only one out of 17 KEGG pathways, *Axon guidance*, discriminating between adenomas and adenocarcinomas (Table 5), has been found significant for the GSE4183 data set (Table S7). On the other hand, several pathways extracted by our procedure from the GSE4183 data set were common with the KEGG pathways distinguishing between colonic epithelial cells and mucosa in our data sets (Table 5, and Table S7).

**Table 5:** KEGG terms corresponding to genes differentially expressed between adenomas and carcinomas.

Whole tissue sections
Adherens junction
p53 signaling pathway
PPAR signaling pathway
Wnt signaling pathway
Calcium signaling pathway
TGF-beta signaling pathway
Axon guidance
Colorectal cancer
Pancreatic cancer
Bladder cancer
Proteasome
Valine, leucine, and isoleucine degradation
Fatty acid metabolism
Tryptophan metabolism
Sphingolipid metabolism
Biosynthesis of unsaturated fatty acids
Arachidonic acid metabolism

### Gene expression diagnostic for the progression to CRC

Gene expression signature that can be employed for diagnostic purposes should consist of probe sets with signal progressively increased or decreased over the normal tissue-adenoma-carcinoma sequence. To select CRC progression markers, the procedure was consistently applied to analyze data sets obtained by two tissue handling procedures. Probe sets with gradually increasing or decreasing expression (*p*<0.01, FDR adjusted) throughout CRC progression that were found in all three sample sets (whole tissue sections, microdissected epithelial cells, and microdissected mucosa) are presented in Table 6 and 7, respectively. Of these probe sets, a few were representative for data sets normalized with both algorithms.

**Table 6:** Genes with gradually increasing expression (*p*<0.01, FDR adjusted) through CRC progression in all three sample sets.

Up-regulated	
GCRMA_LVS	MAS5.0
227140_at	227140_at
Tribbles homolog 3 (Drosophila)	Tribbles homolog 3 (Drosophila)
Collagen, type XII, alpha 1	Collagen, type XII, alpha 1
Solute carrier family 39 (zinc transporter), member 10	Solute carrier family 39 (zinc transporter), member 10
Diaphanous homolog 3 (Drosophila)	Diaphanous homolog 3 (Drosophila)
Jub, ajuba homolog (Xenopus laevis)	Jub, ajuba homolog (Xenopus laevis)
Stearoyl-CoA desaturase (delta-9-desaturase)	Stearoyl-CoA desaturase (delta-9-desaturase)
p53 and DNA damage-regulated 1	p53 and DNA damage-regulated 1
Collagen, type IV, alpha 1	Aminopeptidase-like 1
Collagen, type I, alpha 2	
Hypothetical LOC541471	
General transcription factor IIIA	
Nnicotinamide N-methyltransferase	
Phosphoprotein enriched in astrocytes 15	
Solute carrier family 7 (cationic amino acid transporter, y+ system), member 5	
Chromosome 13 open reading frame 3	
Core-binding factor, beta subunit	
Regulator of chromosome condensation (RCC1) and BTB (POZ) domain containing protein 1	
RecQ protein-like (DNA helicase Q1-like)	
Glucosamine-6-phosphate deaminase 1	
Proteasome maturation protein	
Breast cancer 2, early onset	
Ubiquitin D	
Nuclear factor (erythroid-derived 2)-like 3	
Phosphoglucomutase 3	
Tryptophanyl-tRNA synthetase	
CCAAT/enhancer binding protein (C/EBP), beta	
Hypothetical protein MGC15523	
Calumenin	
Transmembrane protease, serine 3	
Chemokine (C-X-C motif) ligand 11	

Probe set names are given where no gene name is available.

**Table 7:** Genes with gradually decreasing expression (*p*<0.01, FDR adjusted) through CRC progression in all three sample sets.

Down-regulated	
GCRMA_LVS	MAS5.0
Hypothetical protein FLJ21511 (220724_at; 220723_s_at)	Hypothetical protein FLJ21511 (220724_at; 220723_s_at)
Protein kinase, cAMP-dependent, catalytic, beta	Protein kinase, cAMP-dependent, catalytic, beta
Hypothetical protein LOC253012	Hypothetical protein LOC253012
protein kinase, cAMP-dependent, catalytic, beta	Protein kinase, cAMP-dependent, catalytic, beta
UDP glucuronosyltransferase 1 family, polypeptide A1	UDP glucuronosyltransferase 1 family,polypeptide A1
Ring finger protein 125	Ring finger protein 125
UDP glucuronosyltransferase 1 family, polypeptide A6	UDP glucuronosyltransferase 1 family, polypeptide A6
B-cell CLL/lymphoma 2	B-cell CLL/lymphoma 2
Zinc finger and BTB domain containing 7C	227630_at
Hypothetical protein LOC92482	Nuclear receptor subfamily 3, group C, member 2
CAS1 domain containing 1	Sterile alpha motif domain containing 13
Somatostatin receptor 1	Similar to all-trans-13,14-dihydroretinol saturase
Abhydrolase domain containing 3	Programmed cell death 4 (neoplastic transformation inhibitor)
Bestrophin 2	N-acetylglucosamine-1-phosphate transferase, alpha and beta subunits
UDP glucuronosyltransferase 1 family, polypeptide A9	

Probe set names are given where no gene name is available.

The level of these probe sets appeared to be significantly altered also in other microarray studies [14]–[16] (Table S7). Nine, 14 and 15 of them were found to significantly differentiate NC and AD or NC and CA (FDR<0.01 and FC>1.5) in two studies conducted by Galamb et al. [16] [15] and Sabates-Bellver et al. [14], respectively.

## Discussion

Carcinogenesis is a microevolutionary process that results from a series of genetic and epigenetic alterations. As a consequence of the successive rounds of mutation and selection of cell clones, molecular alterations affect the fundamental processes of a normal cell, such as proliferation, differentiation, and apoptosis [1]. Though multiple proto-oncogenes and tumor suppressor genes play an essential role in neoplastic growth, no more than 15 somatic “driver” mutations are thought to be responsible for individual cancer initiation, progression, and maintenance. However, among a group of clinically homogenous tumors, only a few mutated “cancer genes” are shared [3], [4]. These highly variable patterns of somatic mutations in cancer genomes are likely responsible for biological differences among cancers. On the other hand, cancers share, at least in part, common phenotypes that lead to standard treatment algorithms.

Most CRCs arise in a progression through adenoma to carcinoma phenotypes as a consequence of altered genetic information. Because genetic information is utilized by macromolecules (RNA and proteins) and metabolites representing short-term storage, selectively provided at the time and grouped into signaling and metabolic pathways, cancer complexity at the gene level is likely reduced to a limited number of altered pathways. Thus, the clinical progression of colon tumor phenotypes may occur in parallel to distinctive signaling alterations [5].

Several microarray-based studies in CRC have been performed [18], [19], most to identify discriminative gene expression profiles for diagnostic and prognostic purposes [17], [20]–[29]. Some other studies were performed to identify molecular processes underlying tumorigenesis and metastasis [30]–[35]. However, a comparative analysis of the above mentioned studies revealed rather weak overlap of catalogued gene expression profiles [7], which is mostly a consequence of microarray experiments generating large sets of data that are not directly interpretable. As a result, significant data pre-processing is required to convert raw data (images of the scanned chips) into meaningful biological knowledge. The analysis of a typical microarray experiment involves the following steps: 1) image processing, 2) probe-level processing aimed at the generation of gene-expression summaries and minimizing technical variability introduced during sample preparation and measurement, 3) statistical analysis that, depending on the scientific goal of the experiment, may be focused on grouping genes with similar expression, identifying genes differentially expressed between two or more experimental conditions, or discovering unknown subclasses of samples correlating with the phenotype or clinical course, and 4) higher level (functional) analysis, which allows for biological interpretation of the results. Notably, no fully acceptable protocol exists for microarray data processing, and the different methods used for subsequent stages of the analysis pipeline may result in substantially different results. Therefore, the final result of microarray studies is a function of not only the biological information in the samples, but of the choices made during data processing. The problem of an extensive data transformation results in a situation in which we cannot always justify the biological meaning of a particular readout, and adjustment of the final results to the working hypothesis may introduce systemic bias and, in the end, manipulate the results.

We aimed to identify the essential oncogenic signaling in CRC. Because there is no method that allows the direct estimation of changes in cellular signaling on the genomic scale, we applied an integrative genomics approach that may connect gene expression profiles with molecular pathway alterations. The assessments were directed to minimize the number of prior assumptions and arbitrary choices made during data processing, particularly in the two steps known to have the most severe impact on the results of the analysis: probe-level processing and selection of differentially expressed genes.

Several probe-level processing methods have been proposed, including MAS5.0 [36], MBEI [37], RMA [38], and GCGMA [39]. Although this stage of the analysis pipeline is often simply referred to as normalization, it usually involves three separate steps: background adjustment, normalization, and summarization. Background adjustment is aimed at removing the influence of the optical noise, autofluorescence of the chip surface, non-specific binding, and cross-hybridization from the measured signal. Next, probe intensities are normalized in order to allow direct comparisons between chips and minimize the technical variation that results from possible differences in total mRNA quantities or unequal efficiencies in labeling and hybridization. Finally, in the summarization step, the adjusted and normalized intensities in each probe set are combined into a single numerical value that represents the relative abundance of a transcript in the sample.

The reliable evaluation of the performance of low-level processing methods is not an easy task because it requires prior knowledge of the data properties, especially which genes are truly differentially expressed. Consequently, most of the published comparisons of low-level processing algorithms rely on an RNA spike-in or dilution datasets [40]–[43]. Because the true expression ratios are known in such datasets, they can easily be used to study the bias (accuracy) and variance (precision) of the gene expression estimations derived by different methods. However, though artificially generated datasets are potentially useful validation tools, there might be some doubt as to whether they actually represent the characteristics of the data from typical microarray experiments in terms of the fraction of differentially expressed genes or RNA quality. Furthermore, the spike-in and dilution data consist of technical replicates, and thus do not reflect the true biological variability between samples from typical data sets. Despite numerous efforts [44], there are still no widely accepted methods for assessing the effectiveness of low-level processing for a particular real-world dataset.

To avoid the arbitrary choices in expression summary generation and data normalization, all presented analyses have been carried out using two algorithms: MAS5.0 and GCRMA combined with LVS normalization [45]. MAS5.0 is a relatively simple algorithm that processes a single chip at a time, utilizing probe position on the chip and mismatch (MM) probes to correct the perfect match (PM) probe signal readout. Normalization is performed after the summarization step by global scaling so that the trimmed mean intensities of the arrays to be compared are identical. GCRMA takes into the account the GC content of the probe and conducts normalization on a full set of microarrays. GCRMA does not utilize the MM probe signal. The LVS algorithm normalizes the data set based on the least variant probe sets and replaces the quantile normalization implemented in GCRMA.

As expected, the use of two different algorithms resulted in data sets with considerably different distributions of normalized relative expression values. Therefore, to maximize reliability, both data sets were used in further analyses. In addition, we filtered out low-expression probe sets that, due to unfavorable signal-to-noise ratio, were likely to be a source of false positives.

Another factor strongly affecting the final results of microarray data analysis is the method used to define differentially expressed genes. A multitude of strategies have been proposed for this task, ranging from simple methods using only the fold change criterion to more sophisticated approaches using permutation-based statistical testing [46] or a Bayesian probabilistic framework [47]. However, with most of the methods it is necessary to specify strict criteria a gene must meet to be considered differentially expressed, for example, FDR thresholds and/or fold change. Various criteria produce substantially different lists of genes as the basis of further biological conclusions. Furthermore, such methods assume an unambiguous assignment of the samples to the classes representing experimental conditions, which is not always the case. The existence of subclasses with different expression profiles (e.g., a tumor with low amount of carcinoma) may alter the feature selection process and mislead further functional analyses of differentially expressed genes.

In order to overcome the mentioned limitations of typical feature selection procedures, we used an approach that combines supervised statistical testing with an unsupervised method, which does not require prior knowledge of sample attribution.

The method selected for the functional analysis used the K-S test. In contrast to common over-representation methods, the test does not require arbitrary cut-off criterion for studied probe sets. The significance of gene sets is determined based on their position on a sorted list of all genes present in the assay. Here, sorting was based on either the *p*-values of a statistical test in a pair-wise comparison (supervised method) or the contribution to a selected SVD component (unsupervised method). Such methodology requires only one arbitrary cut-off: the adjusted *p*-value designating the significance of a given gene set.

The gene set dictionary selected for analysis was KEGG. Each set consists of the genes involved in a physiological process. Most of the KEGG subsets contain genes that interact with each other; thus, the structure of the database, in contrast to Gene Ontology (GO), is “flat”. No relationship between the specified pathways simplifies the correction for multiple hypothesis testing. The number of categories in the KEGG database is a factor of 100 less than GO (∼200 *vs.* ∼30,000). Therefore, the chance of a false positive result is significantly less when the KEGG database is used and less strict correction for multiple hypothesis testing is required.

In this study, pathways were selected as significant based on a consensus of the results obtained with two normalization algorithms, and two probe set sorting criterions. In whole tissue sections we identified 14 and 17 KEGG signaling and metabolic pathways significantly altered between normal colon and colon tumors and between benign and malignant tumors, respectively. Altogether, cell proliferation and differentiation, the regulation of gene expression, DNA repair, cell growth and survival, the signaling (TGF-beta, Wnt, PPAR, Calcium) pathways, aminoacids and lipids metabolism may be considered the predominant alterations, appearing on different levels of molecular interaction and reaction networks of oncogenic signaling in the colon.

Using microdissected tissues, a considerable set of pathways have been selected which differentiate between epithelia and mucosa, regardless of the disease stage. Of these pathways, a wide array of biological processes, including antigen processing and presentation, immune response, and adaptive inflammatory host defenses, cell migration, cell-cell and cell-matrix adhesion, clearly differentiated functions attributed to cells forming the epithelial layers from those of the resident immune cells infiltrating the lamina propria. Several KEGG pathways distinguishing between microdissected colonic epithelial cells and mucosa were also selected from comparisons between whole tissue sections of adenomas and adenocarcinomas (Table S6).

Results of NC - AD/CA comparisons in our dataset are, in general, in good concordance with such comparisons performed on datasets provided by Galamb et al. (GSE4183) [15] and Sabates-Bellver et al. (GSE8671) [14]. On the other hand, there is little overlap between the former of those studies and our dataset for AD - CA comparison. Notably, several pathways found as relevant in GSE4183 dataset were found to be differentiating epithelia and mucosa (Table 5) in our study. However, paper by Galamb et al. does not provide details on sample cellular composition and without an access to the histological assessment of tissues enrolled to microarray studies further result comparison between different studies may be possible only to some extent.

There are over 80 KEGG pathways found significant when all comparisons from publicly available data are taken into account. This, and the fact that there are no "standard" protocols for functional analysis of microarray experiments could lead to tuning the analysis procedure to fit the expected results. We regard that such pitfall could be avoided by selecting the intersection of results acquired with multiple protocols as proposed in this study.

To date, a variety of methods have been used to dissect the tissue of interest, as well as RNA extraction and amplification protocols and algorithms for data normalization and significant features selection. Thus, any variation in the procedure introduces difficulties in the direct comparison of the results. Consistent with this idea, probe sets with the same signal level alterations, regardless of the sample handling protocol applied, are of particular interest. Genes found to be differentially expressed between normal colon, adenoma, and carcinoma can be used as markers of the progression process. If the differences are progressive, the interpretation of the results is straightforward; the higher the difference detected, the more advanced the carcinogenesis.

We found 17 probe sets with both attributes: significant (FDR<0.01) progressive signal level changes in the NC → AD → CA sequence for microdissection and whole tissue section collected samples, and for MAS5.0 and GCRMA+LVS normalization (Table 6 and 7). Eight of the probe sets were progressively up-regulated (Table 6) and nine were down-regulated (Table 7). Expression of most of these probe sets were also found to be changed in other microarray studies [14]–[16].

In summary, microarray-based gene expression profiles were applied to describe gene regulatory networks appearing on different levels of molecular interaction and reaction networks forming oncogenic signaling in the colon. Although some of the KEGG pathways selected in our analyses may result from differences in the proportion of epithelial and stromal cells excised from adenomas and carcinomas, these studies highlighted significant differences in the molecular makeup of adenomas and adenocarcinomas related to oncogenic signaling. However, though the changes in patterns of individual probe sets annotated to defined KEGG signaling pathways intuitively fit predictions, the microarray data could be translated into the functional aspects of carcinogenesis only as indirect annotations.

The lack of independent methods of verification of functional annotations to expression profiles makes the final conclusions from microarray readouts prone to the subjective selection of biostatistical tools. We proposed to tackle this problem by using a wide range of computational methods. Although this strategy is computationally complex and labor–intensive, it reduces the fraction of false results. Herein, we provided an example of such a multidirectional algorithm directed for maximizing the reliability of microarray data results. On the other hand, with such strict conditions, only highly reliable biological processes are selected. Although many pathways may be missed this way, the low concordance of published results justifies the applied conditions.

The main disadvantage of the proposed functional analysis relates to the assumption that the number of affected genes within a single pathway determines the degree of its alteration. This assumption may be correct for most metabolic pathways that rely on the mass-action law [48] but may not hold true for signaling pathways. In fact, the universal number of genes altered in a single pathway to consider the pathway as affected remains unknown. Consequently, the lack of knowledge about the compensation effect and its extent makes such analyses incomplete. If so, the full discovery and understanding of biological processes underlying CRC on a genomic scale would be possible only with a more stringent approach, which requires an extensive knowledge of signaling mechanisms in all biological processes considered. This approach is not feasible with the currently available bioinformatics tools.

## Materials and Methods

### Ethics Statement

Patients were prospectively selected for the study between January and December 2006 at the Department of Gastroenterology and Hepatology and the Department of Colorectal Cancer, Cancer Center-Institute, Warsaw. The study protocol was approved by the Cancer Center Bioethics Committee, and all patients signed informed consent before inclusion.

Sporadic colonic carcinomas were obtained by surgical resection through laparotomy, and one to four tumor fragments, depending on the tumor's size, and two fragments of paired full-thickness normal colon were cut. Colonic adenomas were obtained during colonoscopic polypectomy, and one to three adenoma fragments, depending on the polyp's size (0.8–3.0 cm), were cut from the tip of each polyp immediately after removal. The amount of removed polyp did not interfere with the histological diagnosis. All carcinoma and polyp tissue specimens were collected by pathologists using the same procedure. In addition, three biopsies were taken from the normal colonic mucosa of 7 healthy subjects who underwent screening colonoscopy using large biopsy forceps. All tissue specimens were snap frozen in liquid nitrogen within 10–30 min of harvesting and stored at −72°C until use.

The clinical characteristics of patients and histopathology of analyzed tissue samples are presented in Table S8.

### Microdissected samples

Frozen tissue specimens were cut as a series of 6-µm thick cryosections and mounted on a polyethylene naphthalate (PEN) membrane slide and dehydrated for 2 min in 70% ethanol and 5 min in 100% ethanol. Subsequently, tissue sections were stained in 5% (w/v) alcoholic solution of cresyl violet, rinsed in 100% ethanol, and the slides air-dried for 15 min. The interested areas were independently isolated from the slides using the PALM laser microdissection and pressure catapulting (LMPC) system (PALM MicroBeam with PALM RoboMover module and PALM RoboSoftware; Carl Zeiss MicroImaging GmbH, Germany). Microdissected samples from different parts of the tumor and normal colon were pooled in separate microtubes, immediately lysed with 100 ml RTL buffer (Qiagen GmbH, Hilden, Germany) containing 1% β-mercaptoethanol, and stored at −72°C.

To obtain suitable reproducibility and reliability of the estimations, tissue samples were dissected by LCM of five replicates for each type of epithelial cell and mucosa from one colon tumor and the paired full-thickness normal colon. Independent microdissections of epithelial cell layers yielded an average of 2.8 mm^2^ of total captured area (range 2.1–3.5 mm^2^), whereas captured mucosa that represented a normal or neoplastic epithelial layer with an absent lamina propria of the muscularis mucosae yielded an average 13 mm^2^ of total area (range 10–15 mm^2^). The relative cell type content within normal and dysplastic mucosa estimated per 1 mm^2^ is presented in Table 1.

### Whole tissue sections

Several series of cryostat sections were prepared from different parts of each specimen using a Microm HM 505E (Zeiss, Germany). Upper and lower sections from each cryosection collection were evaluated by the pathologist to control the relative cell type content. RNA was isolated from those cryostat sections representing a given tissue specimen which contained the highest percentage of epithelial cells.

Histological evaluation of the examined tissues revealed a median relative content of 60% (range 18–98%) normal mucosa in surgically obtained normal colon specimens and 90% (28–99%) and 55% (15–98%) dysplastic mucosa in specimens representing benign and malignant colon tumors, respectively (Table S8). Endoscopic biopsies from the normal colons represented mostly mucosa. Thirty-one polyps were identified as tubular adenomas, and 14 as tubulo-villous adenomas; 42 and 3 adenomas exhibited low-grade and high-grade dysplasia, respectively. Altogether, the whole tissue section samples represented 45 colon adenomas, 36 adenocarcinomas, and 24 normal colon samples, of which 7 were obtained during screening colonoscopies, 14 were taken from the full-thickness normal colon at least 5 cm distant from adenocarcinoma, and 3 represented normal mucosa directly adjacent to the neoplastic tissues.

### RNA extraction and amplification

Total RNA was isolated from whole tissue sections and microdissected tissue samples using the RNeasy Plus Mini Kit and QIAshredder columns and the RNeasy Plus Micro Kit (Qiagen GmbH, Hilden, Germany), respectively. RNA samples were checked for quality on the Agilent 2100 Bioanalyzer. Each sample used for further microarray analysis presented distinct peaks corresponding to intact 28S and 18S ribosomal RNA.

Five micrograms of total RNA isolated from each whole tissue section sample and 10–50 ng of total RNA isolated from microdissected samples were used as starting material for the synthesis of biotin-labeled cRNA with one and two rounds of amplification using One-cycle and Two-cycle Target Labeling and Control Reagents (Affymetrix), respectively, the latter with the MEGAscript High Yield Transcription Kit (Ambion Inc, Austin USA). The biotin-labeled cRNA was purified using RNeasy spin columns, fragmented, and hybridized on Affymetrix oligonucleotide microarrays (GeneChip HG-U133plus2).

### Gene expression microarray analysis

To measure gene expression, probe set data (cell intensity files) were generated using two standard normalization algorithms: Affymetrix Microarray Suite v.5 (MAS5.0) and GCRMA with least-variant set (LVS) probe sets. The calculations were performed using R/BioConductor (version 2.8.1) packages affy (version 1.20.2), gcrma (version 2.14.1), and FLUSH.LVS.bundle (version 1.2.1, proportion = 0.6). For data filtration, we selected the probe sets with signal intensity above the threshold limit in at least 5% of samples. The threshold was established at the 98th percentile of the expression levels from Y-chromosome–linked probe set signals detectable in female samples. In addition, the probe sets with signal FC higher than 1.5 (in relation to median) in less than 6 samples were removed from whole tissue sections dataset.

To establish differences in gene expression between tissue groups, both gene-by-gene statistical testing using the permutation test with t statistics and singular value decomposition (SVD) of the data matrix were employed. Because experimental variability is common among microarray data sets, the *p*-values were adjusted for multiple hypothesis testing using the Benjamini-Hochberg procedure [49] to control the false discovery rate (FDR). Probe sets sorted according to either the adjusted *p*-values of the t-test or the contribution of a selected SVD mode were assigned to Kyoto Encyclopedia of Genes and Genomes (KEGG) Pathways.

The one-sided Kolmogorov-Smirnoff test (KS test) was used to calculate whether probe sets attributed to a particular signaling pathway located closer to the top of the lists than expected by chance. Next, the resulting *p-*values were corrected for testing multiple hypotheses. We considered the alterations in KEGG pathways as most significant if the adjusted KS test *p-*values derived from both orderings (by the pair wise test statistic and SVD) were less than 0.01 in at least one data set normalized by either the MAS5.0 or GCRMA+LVS algorithm, or if the adjusted *p-*value was less than 0.05 in both data sets.

Statistical and functional analyses were performed using proprietary software working in MATLAB (R2009a, MathWorks) and R/Bioconductor environments.

The same workflow was applied for external datasets: GSE4183 and GSE8671. The threshold for data filtration was established on the same level as for our “whole tissue section” dataset. Probe sets with signal level higher that threshold in less than 5 and 6 samples (GSE4183, GSE8671 respectively) were filtered out.

### Supplementary data

Supplementary data and all MIAME compliment microarray data are available at Gene Expression Omnibus (GSE20916).

http://www.ncbi.nlm.nih.gov/geo/query/acc.cgi?token=bhervmwuqemiyra&acc=GSE20916

## Supporting Information

Figure S1 Distribution of probe set signals. (A,B) Histograms of signals extracted from microdissected samples using MAS5.0 and GCRMA+LVS, respectively. (C,D) Histograms of signals extracted from macrodissected samples using MAS5.0 and GCRMA+LVS, respectively.
(0.18 MB PDF)

Figure S2 Diagrams of the first SVD modes representing macrodissected adenomas (red) and carcinomas (blue); data normalized by MAS5.0 (left panels) and GCRMA+LVS (right).
(0.05 MB PDF)

Figure S3 Venn diagram presenting numbers of probe sets differentiating normal colon (NC) mucosa and adenoma (AD) (left panel) or normal colon and colorectal cancer (CRC) (right panel) in a given studies. Data was normalized with GCRMA+LVS. Difference was considered significant if FDR in permutation test was less than 0.05.
(0.06 MB PDF)

Table S1 Summary of the quality parameters of individual arrays. Analyzed parameters were established by Affymetrix for GeneChip hybridization with cRNA synthesized by one- or two-cycle amplification procedures.
(0.28 MB DOC)

Table S2 Significant KEGG terms selected by K-S test according to lists of probe sets sorted by p-value in pair-wise comparisons of pure colonic crypt epithelial cells (CEC) and mucosa (MUC) dissected from normal colon (NC), normal mucosa adjusted to neoplastic tissue (NT), adenoma (AD) and carcinoma (CA).
(0.06 MB DOC)

Table S3 Lists of the probe sets sorted either by contribution to a selected SVD component or p-value in pair-wise comparisons for the whole tissue section samples. Provided as zipped excel file.
(3.46 MB ZIP)

Table S4 The summary of the significance of differential representation of KEGG terms according to lists sorted by contribution to a selected SVD component or p-value in pair-wise comparisons of microarray data of whole tissue section samples.
(0.07 MB DOC)

Table S5 Probe sets sorted according to the p-value in pair-wise comparisons between benign and malignant whole tissue sections of colonic neoplasms.
(3.59 MB XLS)

Table S6 Summary of the significance of the differential representation of KEGG pathways selected by the K-S test from probe sets lists sorted either by contribution to a selected SVD component or p-value in pair-wise comparison of whole tissue sections of adenoma (AD) and carcinoma (CA) samples.
(0.07 MB DOC)

Table S7 Significant KEGG pathways for comparing normal colon samples with colonic neoplasms and adenomas with adenocarcinomas in GSE4183 and GSE8671 datasets.
(0.03 MB XLS)

Table S8 Patient clinical characteristics and histopathology of analyzed tissue samples.
(0.18 MB DOC)
