# Correction: Modeling Oncogenic Signaling in Colon Tumors by Multidirectional Analyses of Microarray Data Directed for Maximization of Analytical Reliability

**DOI:** 10.1371/annotation/8c585739-a354-4fc9-a7d0-d5ae26fa06ca

**Published:** 2010-12-02

**Authors:** Magdalena Skrzypczak, Krzysztof Goryca, Tymon Rubel, Agnieszka Paziewska, Michal Mikula, Dorota Jarosz, Jacek Pachlewski, Janusz Oledzki, Jerzy Ostrowski

The ninth author's name was misspelled. The correct name is: Jerzy Ostrowski. 

