# Supplementary figures and images for: Modeling Oncogenic Signaling in Colon Tumors by Multidirectional Analyses of Microarray Data Directed for Maximization of Analytical Reliability
Source: PLoS One. 2010 Oct 1;5(10):e13091. doi: 10.1371/journal.pone.0013091 (PMC2948500; doi:10.1371/journal.pone.0013091)

**Figure S1**

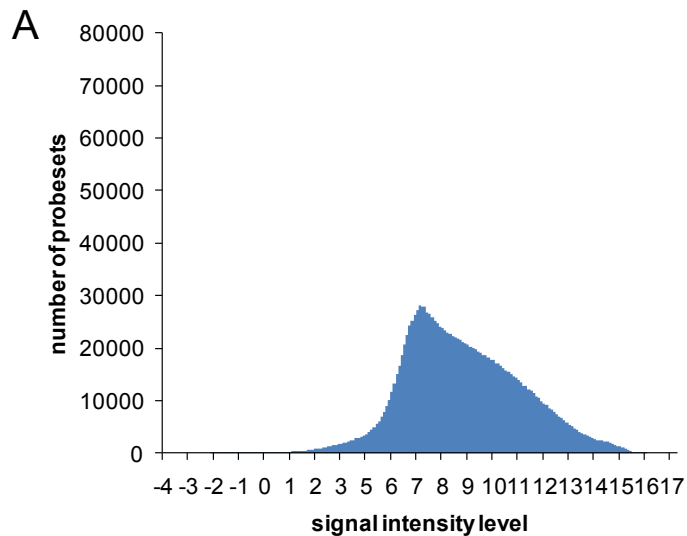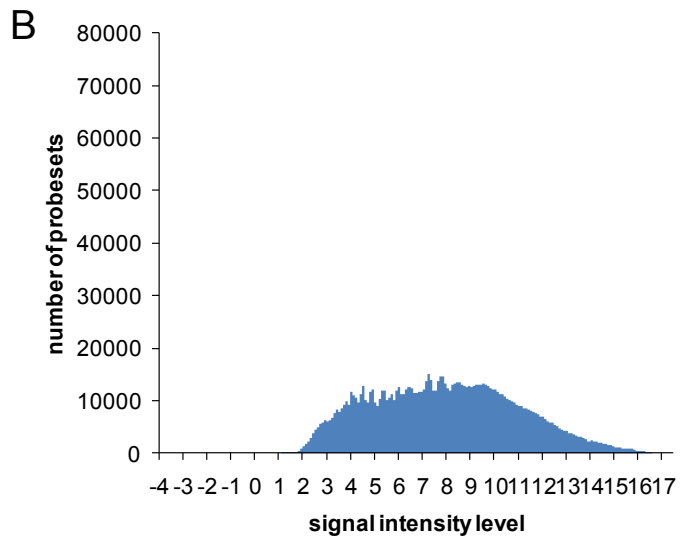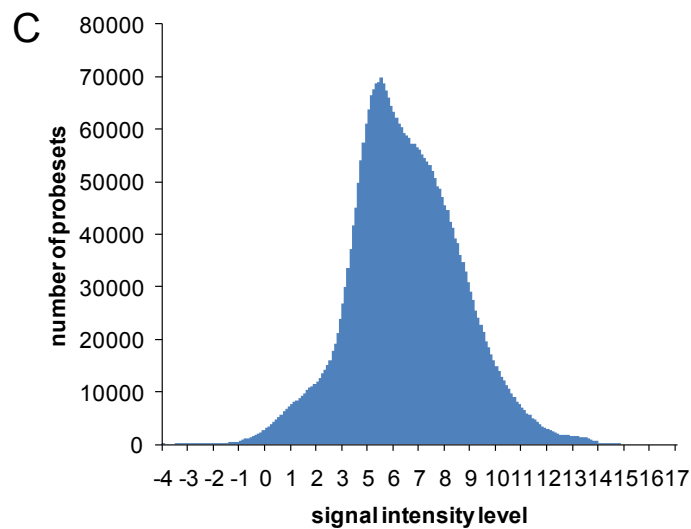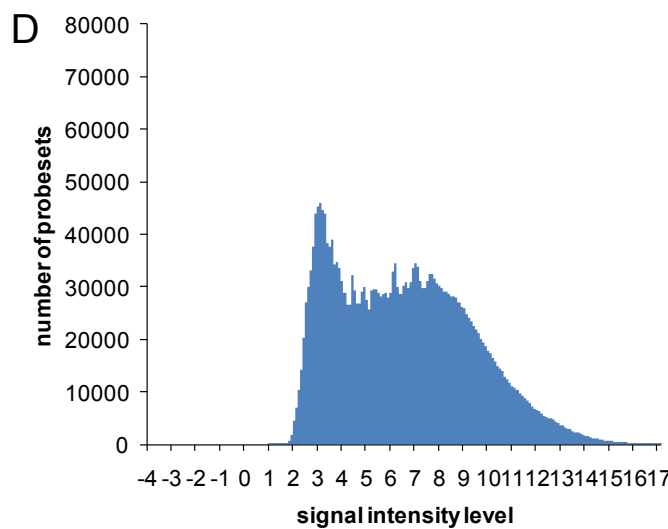

Supplement: Figure S1 — Distribution of probe set signals. (A,B) Histograms of signals extracted from microdissected samples using MAS5.0 and GCRMA+LVS, respectively. (C,D) Histograms of signals extracted from macrodissected samples using MAS5.0 and GCRMA+LVS, respectively. (0.18 MB PDF) [file pone.0013091.s001.pdf]

**Figure S2**

MAS5.0

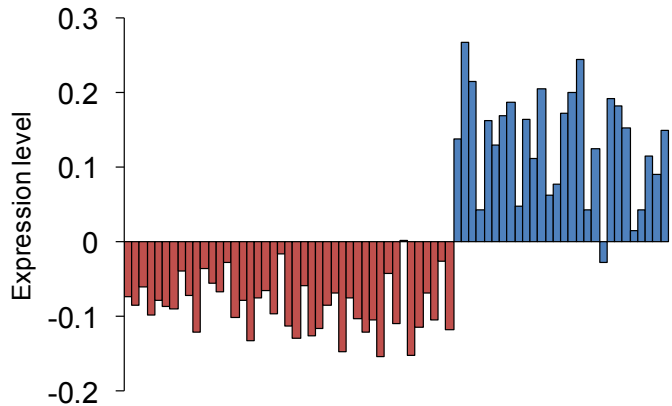

GCRMA+LVS

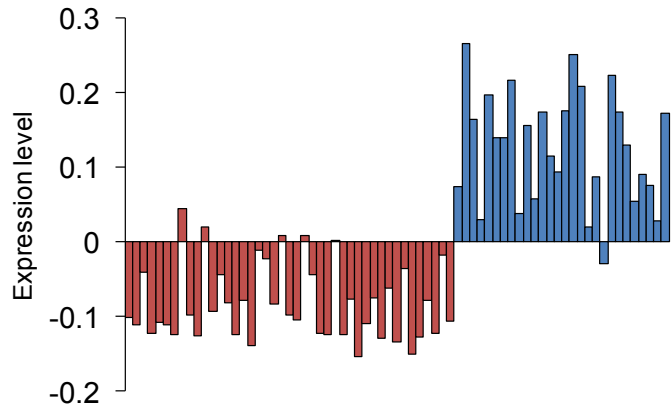

Supplement: Figure S2 — Diagrams of the first SVD modes representing macrodissected adenomas (red) and carcinomas (blue); data normalized by MAS5.0 (left panels) and GCRMA+LVS (right). (0.05 MB PDF) [file pone.0013091.s002.pdf]

**Figure S3**

NC – AD, FDR<0.05

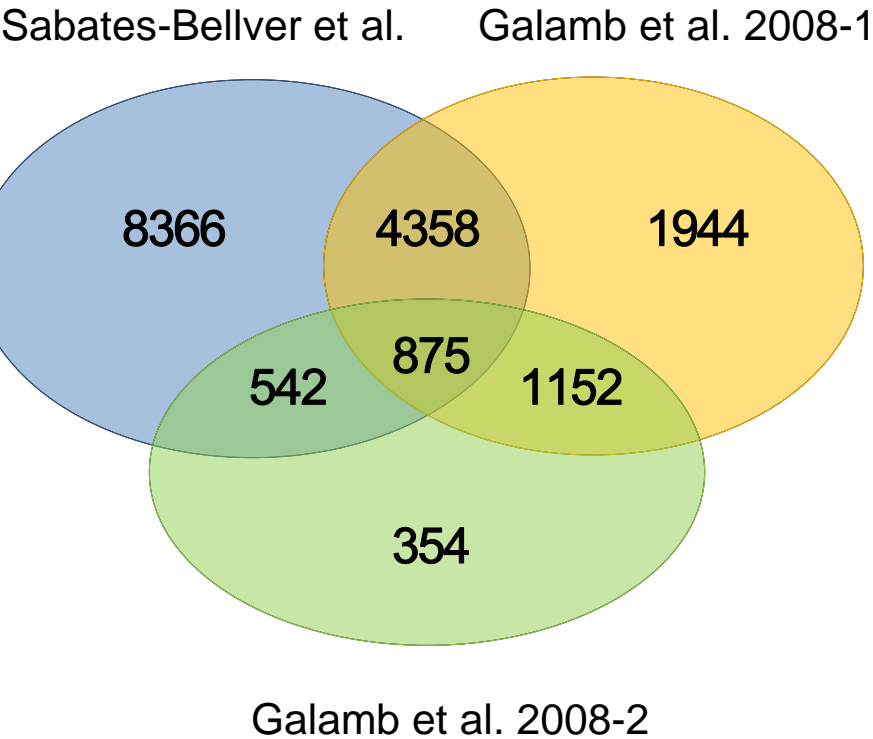

NC – CRC, FDR<0.05

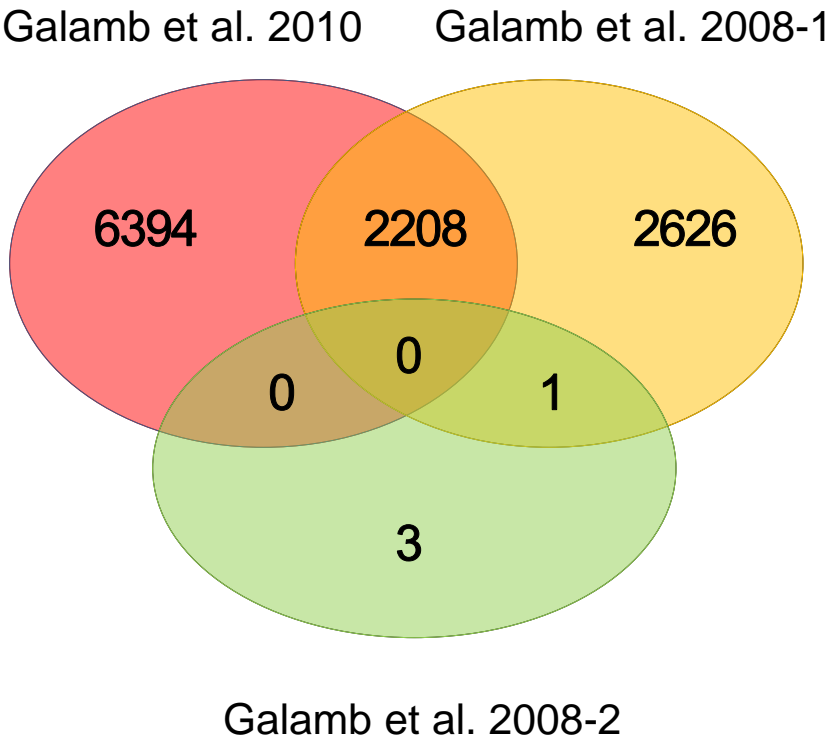

Supplement: Figure S3 — Venn diagram presenting numbers of probe sets differentiating normal colon (NC) mucosa and adenoma (AD) (left panel) or normal colon and colorectal cancer (CRC) (right panel) in a given studies. Data was normalized with GCRMA+LVS. Difference was considered significant if FDR in permutation test was less than 0.05. (0.06 MB PDF) [file pone.0013091.s003.pdf]
