# Supplementary material for: Modeling Oncogenic Signaling in Colon Tumors by Multidirectional Analyses of Microarray Data Directed for Maximization of Analytical Reliability
Source: PLoS One. 2010 Oct 1;5(10):e13091. doi: 10.1371/journal.pone.0013091 (PMC2948500; doi:10.1371/journal.pone.0013091)
Supplement: Table S1 — Summary of the quality parameters of individual arrays. Analyzed parameters were established by Affymetrix for GeneChip hybridization with cRNA synthesized by one- or two-cycle amplification procedures. (0.28 MB DOC) [file pone.0013091.s004.doc]

**Supplementary Table 1**. Summary of the quality parameters of individual arrays. Analyzed parameters were established by Affymetrix for GeneChip hybridization with cRNA synthesized by one- or two-cycle amplification procedures.

| # | Microarray ID | SF | PERCENT | BACKGROUND | BACT | GAPDH | BioB_call | BioB | BioC | BioD | CreX |
| --- | --- | --- | --- | --- | --- | --- | --- | --- | --- | --- | --- |
| 1 | ColonCa_Series01_18A.CEL | 1.49 | 42.28 | 49.3 | 0.53 | 0.21 | P | 7.58 | 8.95 | 11.21 | 13.18 |
| 2 | ColonCa_Series01_18B1.CEL | 1.73 | 39.51 | 48.43 | 1.49 | 0.46 | P | 8.02 | 9.47 | 11.93 | 13.55 |
| 3 | ColonCa_Series01_24B1.CEL | 2.07 | 38.25 | 48.81 | 1.1 | 0.41 | P | 7.81 | 9.53 | 11.94 | 13.61 |
| 4 | ColonCa_Series01_42.6B2.CEL | 1.5 | 43.12 | 51.55 | 1.09 | 0.49 | P | 7.62 | 9.15 | 11.28 | 13.29 |
| 5 | ColonCa_Series01_8K.CEL | 2.71 | 38.83 | 48.55 | 1.69 | 0.38 | P | 8.71 | 9.93 | 12.15 | 14.1 |
| 6 | ColonCa_Series01_P1.CEL | 1.4 | 40.51 | 50.78 | 0.61 | 0.19 | P | 7.15 | 8.96 | 11.07 | 13.1 |
| 7 | ColonCa_Series01_P5D.CEL | 1.22 | 41.05 | 56.77 | 0.58 | 0.19 | P | 7.24 | 8.7 | 10.99 | 12.93 |
| 8 | ColonCa_Series02_11B1N_2.CEL | 2.1 | 40.98 | 52.32 | 0.71 | 0.25 | P | 8.44 | 9.74 | 12.37 | 13.9 |
| 9 | ColonCa_Series02_1K.CEL | 1.72 | 42.29 | 51.21 | 1.17 | 0.69 | P | 8.1 | 9.67 | 11.83 | 13.74 |
| 10 | ColonCa_Series02_27A.CEL | 1.64 | 41.91 | 49.32 | 1.1 | 0.38 | P | 7.55 | 9.34 | 11.59 | 13.51 |
| 11 | ColonCa_Series02_27B1.CEL | 1.31 | 41.02 | 49.89 | 0.63 | 0.15 | P | 7.62 | 9.23 | 11.33 | 13.23 |
| 12 | ColonCa_Series02_46BII6D.CEL | 1.72 | 39.72 | 49.37 | 0.99 | 0.32 | P | 8.03 | 9.6 | 11.81 | 13.6 |
| 13 | ColonCa_Series02_P2.CEL | 1.24 | 43.3 | 50.77 | 1.09 | 0.42 | P | 7.78 | 9.17 | 11.49 | 13.28 |
| 14 | ColonCa_Series02_P6.CEL | 1.83 | 39.3 | 51.68 | 1.55 | 0.6 | P | 8.43 | 10.02 | 12.2 | 13.98 |
| 15 | ColonCa_Series03_14B2.CEL | 2.19 | 40.05 | 55.36 | 1.88 | 0.77 | P | 8.52 | 9.99 | 12.23 | 14.11 |
| 16 | ColonCa_Series03_22A_2.CEL | 2.09 | 39.46 | 53.42 | 1.16 | 0.01 | P | 8.51 | 9.9 | 12.25 | 13.88 |
| 17 | ColonCa_Series03_22B1.CEL | 1.97 | 36.82 | 44.13 | 1.09 | 0.24 | P | 7.68 | 9.4 | 11.41 | 13.59 |
| 18 | ColonCa_Series03_2K.CEL | 1.85 | 41.97 | 48.55 | 1.56 | 0.75 | P | 8.08 | 9.65 | 11.84 | 13.69 |
| 19 | ColonCa_Series03_P7.CEL | 1.24 | 41.58 | 51.25 | 1.22 | 0.34 | P | 7.59 | 9.25 | 11.32 | 13.26 |
| 20 | ColonCa_Series03_P8.CEL | 1.65 | 41.44 | 51.45 | 0.75 | 0.12 | P | 7.69 | 9.38 | 11.55 | 13.43 |
| 21 | ColonCa_Series03_P9A.CEL | 1.98 | 38.05 | 47.32 | 1 | 0.02 | P | 8.04 | 9.68 | 12 | 13.67 |
| 22 | ColonCa_Series04_15B1.CEL | 2.25 | 39.81 | 53.44 | 1.66 | 0.61 | P | 8.21 | 9.64 | 12.06 | 13.93 |
| 23 | ColonCa_Series04_33A.CEL | 1.98 | 38.66 | 49.74 | 0.92 | 0.43 | P | 7.96 | 9.63 | 11.82 | 13.67 |
| 24 | ColonCa_Series04_33B2.CEL | 1.61 | 39.78 | 43.86 | 0.79 | -0.02 | P | 7.66 | 9.3 | 11.5 | 13.33 |
| 25 | ColonCa_Series04_45B1.CEL | 1.73 | 40.64 | 48.36 | 0.54 | 0.3 | P | 7.84 | 9.44 | 11.46 | 13.4 |
| 26 | ColonCa_Series04_P10.CEL | 1.79 | 40.21 | 50.43 | 1.14 | 0.27 | P | 7.73 | 9.18 | 11.29 | 13.23 |
| 27 | ColonCa_Series04_P4.CEL | 1.59 | 39.74 | 49.78 | 0.91 | 0.21 | P | 7.75 | 9.3 | 11.38 | 13.36 |
| 28 | ColonCa_Series04_P9BD.CEL | 2.2 | 35.64 | 47.57 | 1.33 | 0.19 | P | 7.61 | 9.61 | 11.85 | 13.66 |
| 29 | ColonCa_Series05_36B1N.CEL | 2.33 | 40.07 | 46.27 | 0.78 | 0.27 | P | 7.73 | 9.53 | 11.84 | 13.64 |
| 30 | ColonCa_Series05_41A.CEL | 1.27 | 41.38 | 54.56 | 0.65 | 0.32 | P | 7.5 | 9.26 | 11.37 | 13.43 |
| 31 | ColonCa_Series05_41B1.CEL | 2.45 | 36.22 | 49.85 | 1.21 | 0.36 | P | 8.25 | 9.89 | 11.99 | 13.81 |
| 32 | ColonCa_Series05_44B1.CEL | 2.1 | 35.65 | 52.23 | 0.77 | 0.24 | P | 8.68 | 9.94 | 12.16 | 14.01 |
| 33 | ColonCa_Series05_5K.CEL | 2.04 | 39.31 | 48.69 | 1.32 | 0.51 | P | 8.24 | 9.72 | 11.96 | 13.82 |
| 34 | ColonCa_Series05_P13.CEL | 1.61 | 41.84 | 46.26 | 0.76 | 0.25 | P | 8.13 | 9.4 | 11.55 | 13.39 |
| 35 | ColonCa_Series06_15B2.CEL | 1.4 | 45.69 | 49.11 | 0.98 | 0.48 | P | 8.07 | 9.54 | 11.86 | 13.7 |
| 36 | ColonCa_Series06_30B1.CEL | 1.26 | 41.66 | 50.47 | 0.98 | 0.23 | P | 7.87 | 9.44 | 11.76 | 13.49 |
| 37 | ColonCa_Series06_3B1.CEL | 1.1 | 46.04 | 48.74 | 0.82 | 0.1 | P | 7.57 | 9.06 | 11.31 | 13.29 |
| 38 | ColonCa_Series06_P14.CEL | 1.62 | 39.18 | 47.06 | 1.09 | 0.08 | P | 8.03 | 9.6 | 12.2 | 13.91 |
| 39 | ColonCa_Series06_P15.CEL | 1.05 | 40.32 | 54.22 | 1.09 | 0.2 | P | 7.94 | 9.39 | 11.79 | 13.36 |
| 40 | ColonCa_Series06_P19.CEL | 0.99 | 42.69 | 50.65 | 0.81 | 0.13 | P | 7.67 | 9.08 | 11.43 | 13.22 |
| 41 | ColonCa_Series06_P24.CEL | 1.5 | 38.67 | 57.87 | 1.02 | 0.35 | P | 7.94 | 9.41 | 11.98 | 13.59 |
| 42 | ColonCa_Series07_13B1.CEL | 1.13 | 47.23 | 55.94 | 0.85 | 0.32 | P | 7.84 | 9.17 | 11.52 | 13.38 |
| 43 | ColonCa_Series07_29A.CEL | 1.3 | 43.21 | 63.22 | 0.77 | 0.29 | P | 8.11 | 9.52 | 11.88 | 13.65 |
| 44 | ColonCa_Series07_29B1.CEL | 1.37 | 41.86 | 52.27 | 0.83 | 0.31 | P | 8 | 9.5 | 11.8 | 13.62 |
| 45 | ColonCa_Series07_40B2D.CEL | 1.66 | 37.71 | 49.06 | 1.25 | 0.37 | P | 8.31 | 9.88 | 12.2 | 13.81 |
| 46 | ColonCa_Series07_43P.CEL | 1.19 | 42.2 | 54.11 | 0.9 | 0.26 | P | 7.46 | 9.27 | 11.48 | 13.39 |
| 47 | ColonCa_Series07_50PD.CEL | 1.38 | 40.68 | 54.96 | 1.15 | 0.35 | P | 7.77 | 9.43 | 11.71 | 13.54 |
| 48 | ColonCa_Series07_P21.CEL | 1.44 | 39.01 | 47.44 | 0.97 | 0.16 | P | 7.92 | 9.54 | 11.8 | 13.69 |
| 49 | ColonCa_Series08_31A_2.CEL | 1.11 | 42.71 | 59.27 | 0.28 | 0.13 | P | 7.74 | 8.9 | 11.23 | 12.99 |
| 50 | ColonCa_Series08_31BI1_2.CEL | 2.08 | 38.82 | 51.3 | 1.06 | 0.19 | P | 7.87 | 9.69 | 11.86 | 13.88 |
| 51 | ColonCa_Series08_5B1_2.CEL | 1.91 | 37.86 | 49.95 | 1.12 | 0.03 | P | 7.91 | 9.76 | 11.57 | 13.83 |
| 52 | ColonCa_Series08_P16_2.CEL | 1.62 | 39.35 | 55.72 | 0.41 | 0.04 | P | 7.79 | 9.37 | 11.54 | 13.19 |
| 53 | ColonCa_Series08_P17_2.CEL | 1.31 | 38.93 | 55.25 | 1.59 | 0.39 | P | 7.68 | 9 | 11.65 | 13.38 |
| 54 | ColonCa_Series08_P18_2.CEL | 1.64 | 37.1 | 48.58 | 1.26 | 0.22 | P | 7.89 | 9.32 | 11.93 | 13.55 |
| 55 | ColonCa_Series08_P25_2.CEL | 1.51 | 41.18 | 46.38 | 0.7 | 0.26 | P | 8.13 | 9.65 | 11.83 | 13.63 |
| 56 | ColonCa_Series09_38B1.CEL | 1.74 | 39.04 | 46.03 | 1.13 | 0.27 | P | 8.32 | 9.8 | 12.29 | 13.81 |
| 57 | ColonCa_Series09_9A.CEL | 1.72 | 39.92 | 55.78 | 0.93 | 0.25 | P | 8.05 | 9.51 | 11.91 | 13.9 |
| 58 | ColonCa_Series09_9B2.CEL | 1.62 | 43.51 | 46.9 | 0.81 | 0.3 | P | 8.06 | 9.41 | 11.78 | 13.7 |
| 59 | ColonCa_Series09_P20.CEL | 1.22 | 42.81 | 48.18 | 1 | 0.08 | P | 7.92 | 9.33 | 11.69 | 13.35 |
| 60 | ColonCa_Series09_P26.CEL | 1.73 | 37.62 | 51.78 | 0.68 | 0.26 | P | 8.19 | 9.64 | 12.34 | 13.83 |
| 61 | ColonCa_Series09_P29B.CEL | 1.43 | 36.94 | 53.78 | 0.7 | -0.06 | P | 7.97 | 9.26 | 12.01 | 13.32 |
| 62 | ColonCa_Series10_23A.CEL | 1.54 | 43.88 | 46.6 | 1.34 | 0.44 | P | 8.36 | 10.13 | 12.26 | 14.08 |
| 63 | ColonCa_Series10_23B1.CEL | 0.79 | 45.4 | 50.75 | 0.67 | 0.1 | P | 7.59 | 9.09 | 11.37 | 13.14 |
| 64 | ColonCa_Series10_433B1.CEL | 1.19 | 42.04 | 54.85 | 1.01 | 0.2 | P | 7.97 | 9.52 | 11.68 | 13.58 |
| 65 | ColonCa_Series10_P22.CEL | 1.01 | 42.3 | 55.63 | 0.79 | 0.28 | P | 7.74 | 9.31 | 11.39 | 13.2 |
| 66 | ColonCa_Series10_P28C.CEL | 0.88 | 41.08 | 54.93 | 1.22 | 0.23 | P | 7.46 | 8.98 | 11.48 | 13.17 |
| 67 | ColonCa_Series10_P31.CEL | 1.75 | 38.55 | 47.3 | 1.4 | 0.33 | P | 8.39 | 9.86 | 12.28 | 14.02 |
| 68 | ColonCa_Series11_16A.CEL | 2.89 | 36.1 | 47.16 | 1.81 | 0.53 | P | 9.12 | 10.93 | 13.19 | 14.89 |
| 69 | ColonCa_Series11_16B1.CEL | 1.6 | 42.4 | 53.81 | 1.29 | 0.39 | P | 8.5 | 10.03 | 12.23 | 14 |
| 70 | ColonCa_Series11_20B1.CEL | 1.18 | 41.1 | 51.32 | 0.77 | 0.09 | P | 7.96 | 9.44 | 11.89 | 13.56 |
| 71 | ColonCa_Series11_31IIB1.CEL | 0.96 | 43.03 | 57.04 | 0.7 | 0.18 | P | 7.59 | 9.17 | 11.5 | 13.13 |
| 72 | ColonCa_Series11_39P1.CEL | 2.15 | 36.83 | 48.13 | 1.73 | 0.09 | P | 8.96 | 10.35 | 12.71 | 14.47 |
| 73 | ColonCa_Series11_40P.CEL | 1.14 | 42.97 | 54.26 | 0.9 | 0.22 | P | 7.58 | 9.41 | 11.55 | 13.36 |
| 74 | ColonCa_Series11_P33.CEL | 1.38 | 39.27 | 61.84 | 0.84 | 0.42 | P | 8.2 | 9.63 | 11.85 | 13.63 |
| 75 | ColonCa_Series11_P38.CEL | 1.84 | 37.08 | 50.69 | 1.35 | 0.22 | P | 8.2 | 9.78 | 12.06 | 13.96 |
| 76 | ColonCa_Series12_19B2_2.CEL | 1.78 | 38.34 | 55.06 | 0.38 | -0.02 | P | 8.27 | 9.55 | 11.95 | 13.83 |
| 77 | ColonCa_Series12_21A.CEL | 2.11 | 37.46 | 46.43 | 1.68 | 0.34 | P | 8.64 | 10.38 | 12.63 | 14.34 |
| 78 | ColonCa_Series12_21B1_2.CEL | 1.23 | 43.72 | 48.19 | 0.75 | 0.2 | P | 7.62 | 9.3 | 11.54 | 13.36 |
| 79 | ColonCa_Series12_9K.CEL | 1.15 | 43.25 | 54.89 | 0.86 | 0.43 | P | 7.54 | 9.44 | 11.69 | 13.44 |
| 80 | ColonCa_Series12_P23.CEL | 1.62 | 36.51 | 53.71 | 0.97 | 0.09 | P | 8.31 | 10 | 12.36 | 14.03 |
| 81 | ColonCa_Series12_P32A.CEL | 1.35 | 40.76 | 48.91 | 0.95 | 0.27 | P | 7.89 | 9.36 | 11.74 | 13.59 |
| 82 | ColonCa_Series12_P34.CEL | 1.11 | 42.43 | 58.1 | 0.73 | 0.34 | P | 7.9 | 9.46 | 11.5 | 13.48 |
| 83 | ColonCa_Series13_12A.CEL | 1.95 | 40.36 | 47.76 | 1.51 | 0.39 | P | 8.7 | 10.31 | 12.49 | 14.24 |
| 84 | ColonCa_Series13_25B1N.CEL | 1.41 | 39.75 | 47.26 | 0.65 | 0.06 | P | 7.88 | 9.52 | 11.8 | 13.57 |
| 85 | ColonCa_Series13_37B.CEL | 1.1 | 43.94 | 52.36 | 1.3 | 0.54 | P | 7.58 | 9.37 | 11.65 | 13.4 |
| 86 | ColonCa_Series13_41P.CEL | 0.94 | 44.09 | 55.07 | 0.75 | 0.32 | P | 7.6 | 9.09 | 11.37 | 13.22 |
| 87 | ColonCa_Series13_47BIV1.CEL | 1.19 | 41.47 | 51.1 | 1.9 | 0.73 | P | 7.86 | 9.56 | 11.73 | 13.55 |
| 88 | ColonCa_Series13_4K.CEL | 1.21 | 45.73 | 57.44 | 1.2 | 0.42 | P | 7.98 | 9.5 | 11.67 | 13.54 |
| 89 | ColonCa_Series14_1B2.CEL | 1.07 | 45.84 | 53.88 | 1.15 | 0.51 | P | 7.56 | 9.17 | 11.41 | 13.36 |
| 90 | ColonCa_Series14_28B1.CEL | 1.79 | 39.93 | 44.54 | 0.99 | 0.14 | P | 8.15 | 9.85 | 12.08 | 13.89 |
| 91 | ColonCa_Series14_35B2.CEL | 1.99 | 37.23 | 46.66 | 1.61 | 0.51 | P | 7.9 | 10.03 | 12.44 | 14.07 |
| 92 | ColonCa_Series14_3K.CEL | 1.4 | 41.97 | 58.5 | 1.17 | 0.79 | P | 8.19 | 9.71 | 11.86 | 13.75 |
| 93 | ColonCa_Series14_42P.CEL | 1.05 | 44.31 | 54.18 | 0.78 | 0.21 | P | 7.44 | 9.3 | 11.41 | 13.22 |
| 94 | ColonCa_Series14_45P.CEL | 0.99 | 43.8 | 53.89 | 0.78 | 0.3 | P | 7.3 | 9.15 | 11.4 | 13.18 |
| 95 | ColonCa_Series14_47P.CEL | 1.11 | 42.18 | 53.93 | 0.82 | 0.13 | P | 7.62 | 9.11 | 11.39 | 13.22 |
| 96 | ColonCa_Series14_48A.CEL | 1.4 | 41.72 | 56.65 | 1.03 | 0.5 | P | 7.56 | 9.38 | 11.76 | 13.56 |
| 97 | ColonCa_Series15_26B2.CEL | 0.94 | 44.05 | 57.73 | 0.49 | 0.18 | P | 7.34 | 9.25 | 11.39 | 13.11 |
| 98 | ColonCa_Series15_2B2D.CEL | 1.82 | 42.56 | 46.98 | 1.14 | 0.4 | P | 8.51 | 10.26 | 12.49 | 14.17 |
| 99 | ColonCa_Series15_39B2.CEL | 1.36 | 41.49 | 45.16 | 0.72 | -0.02 | P | 8.06 | 9.21 | 11.62 | 13.53 |
| 100 | ColonCa_Series15_48P.CEL | 0.98 | 43.27 | 51.24 | 0.72 | -0.05 | P | 7.02 | 8.88 | 11.16 | 13.05 |
| 101 | ColonCa_Series15_8A.CEL | 2.69 | 37.94 | 42.82 | 1.75 | 0.59 | P | 9.07 | 10.93 | 13.09 | 14.84 |
| 102 | ColonCa_Series15_P27.CEL | 1.34 | 39.58 | 52.86 | 1.14 | 0.41 | P | 8.15 | 9.4 | 11.94 | 13.49 |
| 103 | ColonCa_Series15_P35.CEL | 1.5 | 39.48 | 50.72 | 1.25 | 0.3 | P | 7.65 | 9.61 | 11.8 | 13.57 |
| 104 | ColonCa_Series15_P3.CEL | 1.08 | 42.94 | 51.15 | 0.69 | 0.14 | P | 7.38 | 9.07 | 11.37 | 13.23 |
| 105 | ColonCa_Series17_49P.CEL | 1.35 | 40.18 | 50.41 | 1.05 | 0.25 | P | 7.91 | 9.52 | 11.72 | 13.56 |
| 106 | 11AN.CEL | 1.3495 | 45.46 | 54.44 | 20.25 | 3.934 | P | 7.1044 | 8.8709 | 11.0295 | 12.6012 |
| 107 | 13AN.CEL | 1.3799 | 44.24 | 52.25 | 18.645 | 2.748 | P | 7.4213 | 9.0797 | 11.221 | 12.7559 |
| 108 | 19AA.CEL | 1.3297 | 44.3 | 55.4 | 21.166 | 3.592 | P | 6.9728 | 8.8495 | 11.1064 | 12.6504 |
| 109 | 1EN.CEL | 1.3978 | 45.34 | 44.19 | 72.229 | 11.537 | P | 7.6742 | 9.3337 | 11.5023 | 12.8805 |
| 110 | 20AN.CEL | 1.3969 | 44.16 | 43.72 | 38.24 | 7.218 | P | 7.9543 | 9.6497 | 11.688 | 13.0669 |
| 111 | 21AA.CEL | 1.4161 | 43.49 | 51.02 | 20.327 | 3.749 | P | 7.2189 | 9.1263 | 11.3658 | 12.8863 |
| 112 | 22AC.CEL | 1.5531 | 46.31 | 36.75 | 23.422 | 3.649 | P | 8.0127 | 9.6871 | 11.8158 | 13.3499 |
| 113 | 23AN.CEL | 1.412 | 42.66 | 54.08 | 20.899 | 6.271 | P | 7.5927 | 9.4114 | 11.3746 | 12.8281 |
| 114 | 24AC.CEL | 1.2973 | 47 | 51.07 | 19.764 | 2.812 | P | 7.0553 | 8.8328 | 10.9291 | 12.5004 |
| 115 | 26AC.CEL | 1.2883 | 46.81 | 50.16 | 17.208 | 2.142 | P | 7.0706 | 9.0027 | 10.9303 | 12.4923 |
| 116 | 27AA.CEL | 1.6779 | 40.54 | 38.92 | 39.918 | 4.971 | P | 8.3159 | 10.0549 | 12.2038 | 13.5065 |
| 117 | 28AC.CEL | 1.2629 | 46.71 | 51.55 | 14.318 | 2.126 | P | 7.0535 | 8.7169 | 10.7788 | 12.2863 |
| 118 | 29AA.CEL | 1.3271 | 45.01 | 50.62 | 51.934 | 3.741 | P | 7.4222 | 9.1237 | 11.2912 | 12.7373 |
| 119 | 30AC.CEL | 1.321 | 47.81 | 48 | 21.267 | 3.104 | P | 6.9741 | 8.946 | 11.0551 | 12.6421 |
| 120 | 31EA.CEL | 1.437 | 40.34 | 49.7 | 28.714 | 6.046 | P | 7.7653 | 9.59 | 11.7119 | 13.1371 |
| 121 | 32EN.CEL | 1.441 | 42.47 | 45.07 | 46.801 | 8.946 | P | 7.7636 | 9.5985 | 11.6716 | 13.0976 |
| 122 | 33EA.CEL | 1.4141 | 43.92 | 42.3 | 36.445 | 5.232 | P | 7.7954 | 9.6115 | 11.7177 | 13.0431 |
| 123 | 34EC.CEL | 1.3265 | 44.8 | 55.84 | 37.791 | 7.747 | P | 7.2465 | 9.1213 | 11.1232 | 12.6646 |
| 124 | 35EC.CEL | 1.3509 | 45.55 | 49.84 | 22.479 | 5.275 | P | 7.1113 | 8.8522 | 11.0437 | 12.6386 |
| 125 | 36EA.CEL | 1.3559 | 44.76 | 48.32 | 38.353 | 5.713 | P | 7.1807 | 9.091 | 11.2226 | 12.7384 |
| 126 | 37EN.CEL | 1.4727 | 41.89 | 47.96 | 39.282 | 7.564 | P | 7.6495 | 9.3493 | 11.4006 | 12.9901 |
| 127 | 38EA.CEL | 1.4832 | 42.37 | 45.91 | 45.923 | 8.158 | P | 7.5409 | 9.4113 | 11.5938 | 13.0654 |
| 128 | 39EC.CEL | 1.3694 | 46.06 | 51.11 | 90.824 | 10.546 | P | 7.4382 | 9.1993 | 11.2492 | 12.7788 |
| 129 | 40EN.CEL | 1.4446 | 40.9 | 54.4 | 45.79 | 10.822 | P | 7.6778 | 9.3111 | 11.4242 | 12.862 |
| 130 | 41EA.CEL | 1.3959 | 42.64 | 50.66 | 23.143 | 4.025 | P | 7.5995 | 8.8534 | 11.3139 | 12.7762 |
| 131 | 42EC.CEL | 1.3021 | 47.09 | 49.6 | 46.856 | 6.286 | P | 7.1629 | 9.0132 | 11.1074 | 12.4629 |
| 132 | 43EC.CEL | 1.5008 | 43.73 | 43.36 | 55.254 | 8.478 | P | 8.2595 | 9.7107 | 11.6802 | 13.2501 |
| 133 | 49ENN.CEL | 1.3624 | 43.52 | 54.3 | 48.509 | 17.054 | P | 7.8982 | 9.4153 | 11.5534 | 12.8829 |
| 134 | 50ENN.CEL | 1.5622 | 43.49 | 43.92 | 118.668 | 27.281 | P | 8.2751 | 10.0222 | 12.2298 | 13.4694 |
| 135 | 51ENN.CEL | 1.4533 | 45.02 | 45.93 | 56.772 | 11.226 | P | 7.6356 | 9.3785 | 11.6144 | 13.022 |
| 136 | 52ENN.CEL | 1.5258 | 43.13 | 41.84 | 46.33 | 8.251 | P | 8.114 | 9.7297 | 11.8402 | 13.198 |
| 137 | 53ENN.CEL | 1.4287 | 45.92 | 43 | 55.46 | 7.301 | P | 7.6121 | 9.2702 | 11.4957 | 12.9533 |
| 138 | 54ANN.CEL | 1.4797 | 44.65 | 53.6 | 25.085 | 6.361 | P | 7.5175 | 9.3371 | 11.464 | 12.975 |
| 139 | 55ANN.CEL | 1.4521 | 44.67 | 47.34 | 23.561 | 5.144 | P | 7.432 | 9.4042 | 11.4857 | 13.0625 |
| 140 | 56ANN.CEL | 1.4917 | 45.02 | 42.76 | 43.379 | 6.569 | P | 8.0745 | 9.7993 | 11.9065 | 13.2074 |
| 141 | 58ANN.CEL | 1.3827 | 45.57 | 50.94 | 33.666 | 5.026 | P | 7.6887 | 9.2442 | 11.2933 | 12.8065 |
| 142 | 59ANN.CEL | 1.4602 | 45.36 | 47.67 | 44.337 | 6.128 | P | 7.7329 | 9.372 | 11.5416 | 12.9123 |
| 143 | 64AA.CEL | 1.3246 | 44.63 | 56.89 | 36.764 | 3.904 | P | 7.2569 | 9.0593 | 11.1433 | 12.6937 |
| 144 | 6EN.CEL | 1.4809 | 44.4 | 45.07 | 56.738 | 8.58 | P | 7.5113 | 9.4378 | 11.52 | 12.9691 |
| 145 | 9AN.CEL | 1.3422 | 45.79 | 53.22 | 16.675 | 4.785 | P | 7.2765 | 9.0429 | 11.1599 | 12.7546 |
