# Supplementary material for: Modeling Oncogenic Signaling in Colon Tumors by Multidirectional Analyses of Microarray Data Directed for Maximization of Analytical Reliability
Source: PLoS One. 2010 Oct 1;5(10):e13091. doi: 10.1371/journal.pone.0013091 (PMC2948500; doi:10.1371/journal.pone.0013091)
Supplement: Table S2 — Significant KEGG terms selected by K-S test according to lists of probe sets sorted by p-value in pair-wise comparisons of pure colonic crypt epithelial cells (CEC) and mucosa (MUC) dissected from normal colon (NC), normal mucosa adjusted to neoplastic tissue (NT), adenoma (AD) and carcinoma (CA). (0.06 MB DOC) [file pone.0013091.s005.doc]

**Supplementary Table 2.** Significant KEGG terms selected by K-S test according to lists of probe sets sorted by p-value in pair-wise comparisons of pure colonic crypt epithelial cells (CEC) and mucosa (MUC) dissected from normal colon (NC), normal mucosa adjusted to neoplastic tissue (NT), adenoma (AD) and carcinoma (CA).

| **NC - CEC vs. MUC** | **NT - CEC vs. MUC** | **AD - CEC vs. MUC** | **CA - CEC vs. MUC** |
| --- | --- | --- | --- |
| Cell adhesion molecules (CAMs) | Cell adhesion molecules (CAMs) | Cell adhesion molecules (CAMs) | Cell adhesion molecules (CAMs) |
| ECM-receptor interaction | ECM-receptor interaction | ECM-receptor interaction | ECM-receptor interaction |
| Focal adhesion | Focal adhesion | Focal adhesion | Focal adhesion |
| Allograft rejection | Allograft rejection | Allograft rejection | Allograft rejection |
| Autoimmune thyroid disease | Autoimmune thyroid disease | Autoimmune thyroid disease | Autoimmune thyroid disease |
| Complement and coagulation cascades | Complement and coagulation cascades | Complement and coagulation cascades | Complement and coagulation cascades |
| Asthma | Asthma | Asthma | Asthma |
| Graft-versus-host disease | Graft-versus-host disease | Graft-versus-host disease | Graft-versus-host disease |
| Type I diabetes mellitus | Type I diabetes mellitus | Type I diabetes mellitus | Type I diabetes mellitus |
| Leukocyte transendothelial migration | Leukocyte transendothelial migration | Leukocyte transendothelial migration | Leukocyte transendothelial migration |
| Hematopoietic cell lineage | Hematopoietic cell lineage | Hematopoietic cell lineage | Hematopoietic cell lineage |
| Systemic lupus erythematosus | Systemic lupus erythematosus | Systemic lupus erythematosus | Systemic lupus erythematosus |
| Cytokine-cytokine receptor interaction | Cytokine-cytokine receptor interaction | Cytokine-cytokine receptor interaction |  |
| Neuroactive ligand-receptor interaction | Neuroactive ligand-receptor interaction | Neuroactive ligand-receptor interaction |  |
| Oxidative phosphorylation | Oxidative phosphorylation |  | Oxidative phosphorylation |
| Pathogenic Escherichia coli infection - EHEC | Pathogenic Escherichia coli infection - EHEC |  | Pathogenic Escherichia coli infection - EHEC |
| Antigen processing and presentation | Antigen processing and presentation |  | Antigen processing and presentation |
| Lysosome |  |  | Lysosome |
| Glutathione metabolism |  |  | Glutathione metabolism |
|  | Focal adhesion | Focal adhesion |  |
|  | Vascular smooth muscle contraction | Vascular smooth muscle contraction |  |
|  | Chemokine signaling pathway | Chemokine signaling pathway |  |
|  | Primary immunodeficiency | Primary immunodeficiency |  |
|  | Jak-STAT signaling pathway | Jak-STAT signaling pathway |  |
|  | Arrhythmogenic right ventricular cardiomyopathy (ARVC) | Ether lipid metabolism | Biosynthesis of phenylpropanoids |
|  | Regulation of actin cytoskeleton |  | Biosynthesis of alkaloids derived from histidine and purine |
|  | Basal cell carcinoma |  |  |
|  | Ribosome |  |  |
|  | Natural killer cell mediated cytotoxicity |  |  |
|  | Prion diseases |  |  |
|  | Parkinson's disease |  |  |
