# Supplementary material for: Modeling Oncogenic Signaling in Colon Tumors by Multidirectional Analyses of Microarray Data Directed for Maximization of Analytical Reliability
Source: PLoS One. 2010 Oct 1;5(10):e13091. doi: 10.1371/journal.pone.0013091 (PMC2948500; doi:10.1371/journal.pone.0013091)
Supplement: Table S4 — The summary of the significance of differential representation of KEGG terms according to lists sorted by contribution to a selected SVD component or p-value in pair-wise comparisons of microarray data of whole tissue section samples. (0.07 MB DOC) [file pone.0013091.s007.doc]

**Supplementary Table 4**. The summary of the significance of differential representation of KEGG terms according to lists sorted by contribution to a selected SVD component or p-value in pair-wise comparisons of microarray data of whole tissue section samples.

|  | **SVD1** | **Normal colon *vs.* neoplasms** |
| --- | --- | --- |
| Cell cycle | X | X |
| RNA polymerase | X | X |
| DNA replication | X | X |
| Purine metabolism | X | X |
| Pyrimidine metabolism | X | X |
| Proteasome | X | X |
| p53 signaling pathway | X | X |
| Aminoacyl-tRNA biosynthesis | X | X |
| Nucleotide excision repair | X | X |
| Mismatch repair | X | X |
| Base excision repair | X | X |
| Homologous recombination | X | X |
| Folate biosynthesis | X | X |
| Biosynthesis of unsaturated fatty acids | X | X |
| Ribosome | X |  |
| Oxidative phosphorylation | X |  |
| One carbon pool by folate | X |  |
| Carbon fixation | X |  |
| Biosynthesis of steroids | X |  |
| Vibrio cholerae infection | X |  |
| Fructose and mannose metabolism | X |  |
| Ubiquinone biosynthesis | X |  |
| Pyruvate metabolism | X |  |
| N-Glycan biosynthesis | X |  |
| Benzoate degradation via CoA ligation | X |  |
| Starch and sucrose metabolism | X |  |
| Drug metabolism - other enzymes | X |  |
| Methionine metabolism |  | X |
| Pentose phosphate pathway |  | X |
| Nitrogen metabolism |  | X |
| Glutamate metabolism |  | X |
| PPAR signaling pathway |  | X |
| Apoptosis |  | X |
| Calcium signaling pathway |  | X |
| Cytokine-cytokine receptor interaction |  | X |
| Maturity onset diabetes of the young |  | X |
| Pentose and glucuronate interconversions |  | X |
| Sulfur metabolism |  | X |
| beta-Alanine metabolism |  | X |
| Wnt signaling pathway |  | X |
| Glycerolipid metabolism |  | X |
| Propanoate metabolism |  | X |
| Complement and coagulation cascades |  | X |
| Fatty acid metabolism |  | X |
