# Supplementary material for: Modeling Oncogenic Signaling in Colon Tumors by Multidirectional Analyses of Microarray Data Directed for Maximization of Analytical Reliability
Source: PLoS One. 2010 Oct 1;5(10):e13091. doi: 10.1371/journal.pone.0013091 (PMC2948500; doi:10.1371/journal.pone.0013091)
Supplement: Table S6 — Summary of the significance of the differential representation of KEGG pathways selected by the K-S test from probe sets lists sorted either by contribution to a selected SVD component or p-value in pair-wise comparison of whole tissue sections of adenoma (AD) and carcinoma (CA) samples. (0.07 MB DOC) [file pone.0013091.s009.doc]

**Supplementary Table 6**. Summary of the significance of the differential representation of KEGG pathways selected by the K-S test from probe sets lists sorted either by contribution to a selected SVD component or p-value in pair-wise comparison of whole tissue sections of adenoma (AD) and carcinoma (CA) samples.

| AD *vs.* CA - SVD | AD *vs.* CA –  pair-wise comparison |
| --- | --- |
|  |  |
| **ECM-receptor interaction** | **ECM-receptor interaction** |
| **Cell adhesion molecules (CAMs)** | **Cell adhesion molecules (CAMs)** |
| **Focal adhesion** | **Focal adhesion** |
| **Allograft rejection** | **Allograft rejection** |
| **Autoimmune thyroid disease** | **Autoimmune thyroid disease** |
| **Graft-versus-host disease** | **Graft-versus-host disease** |
| **Type I diabetes mellitus** | **Type I diabetes mellitus** |
| **Leukocyte transendothelial migration** | **Leukocyte transendothelial migration** |
| **Cytokine-cytokine receptor interaction** | **Cytokine-cytokine receptor interaction** |
| Adherens junction | Adherens junction |
| p53 signaling pathway | p53 signaling pathway |
| PPAR signaling pathway | PPAR signaling pathway |
| Wnt signaling pathway | Wnt signaling pathway |
| Calcium signaling pathway | Calcium signaling pathway |
| TGF-beta signaling pathway | TGF-beta signaling pathway |
| Axon guidance | Axon guidance |
| Colorectal cancer | Colorectal cancer |
| Pancreatic cancer | Pancreatic cancer |
| Bladder cancer | Bladder cancer |
| Proteasome | Proteasome |
| Valine, leucine and isoleucine degradation | Valine, leucine and isoleucine degradation |
| Fatty acid metabolism | Fatty acid metabolism |
| Tryptophan metabolism | Tryptophan metabolism |
| Sphingolipid metabolism | Sphingolipid metabolism |
| Biosynthesis of unsaturated fatty acids | Biosynthesis of unsaturated fatty acids |
| Arachidonic acid metabolism | Arachidonic acid metabolism |
|  |  |
| Gap junction | Toll-like receptor signaling pathway |
| Metabolic pathways | Cell Communication |
| Vascular smooth muscle contraction | Fatty acid elongation in mitochondria |
| Arrhythmogenic right ventricular cardiomyopathy (ARVC) | Propanoate metabolism |
| Tight junction | beta-Alanine metabolism |
| Selenoamino acid metabolism | Caprolactam degradation |
| Notch signaling pathway | Butanoate metabolism |
| Regulation of actin cytoskeleton | Drug metabolism - other enzymes |
| Endocytosis | Ascorbate and aldarate metabolism |
| Chemokine signaling pathway | Synthesis and degradation of ketone bodies |
|  | Limonene and pinene degradation |
|  | Cell cycle |
|  | Phenylalanine metabolism |
|  | Neurodegenerative Diseases |
|  | Biosynthesis of steroids |
|  |  |
