# Supplementary material for: Modeling Oncogenic Signaling in Colon Tumors by Multidirectional Analyses of Microarray Data Directed for Maximization of Analytical Reliability
Source: PLoS One. 2010 Oct 1;5(10):e13091. doi: 10.1371/journal.pone.0013091 (PMC2948500; doi:10.1371/journal.pone.0013091)
Supplement: Table S8 — Patient clinical characteristics and histopathology of analyzed tissue samples. (0.18 MB DOC) [file pone.0013091.s011.doc]

**Supplementary Table 8**. Patient clinical characteristics and histopathology of analyzed tissue samples

| # | sex | age | localization | diameter | % of adenoma |
| --- | --- | --- | --- | --- | --- |
| 1 | F | 57 | sigmoid | 12 | 90 |
| 2 | F | 72 | descending | 10 | 68 |
| 3 | F | 57 | sigmoid | 12 | 75 |
| 4 | F | 61 | sigmoid | 9 | 93 |
| 5 | M | 64 | sigmoid | 25 | 93 |
| 6 | M | 80 | transverse | 15 | 95 |
| 7 | F | 60 | descending | 23 | 95 |
| 8 | M | 62 | transverse | 12 | 73 |
| 9 | M | 79 | sigmoid | 20 | 93 |
| 10 | M | 79 | sigmoid | 15 | 95 |
| 11 | M | 81 | transverse | 12 | 90 |
| 12 | F | 68 | sigmoid | 13 | 78 |
| 13 | M | 76 | transverse | 15 | 88 |
| 14 | M | 59 | sigmoid | 8 | 78 |
| 15 | M | 41 | sigmoid | 12 | 88 |
| 16 | M | 55 | sigmoid | 20 | 90 |
| 17 | F | 43 | sigmoid | 22 | 83 |
| 18 | M | 83 | caecum | 15 | 88 |
| 19 | M | 78 | sigmoid | 10 | 88 |
| 20 | M | 60 | sigmoid | 40 | 95 |
| 21 | M | 57 | sigmoid | 12 | 80 |
| 22 | F | 72 | transverse | 13 | 88 |
| 23 | M | 46 | rectum | 20 | 85 |
| 24 | F | 61 | rectum | 15 | 88 |
| 25 | M | 55 | sigmoid | 18 | 88 |
| 26 | M | 52 | ascending | 15 | 58 |
| 27 | F | 75 | caecum | 25 | 90 |
| 28 | M | 68 | sigmoid | 25 | 99 |
| 29 | M | 59 | sigmoid | 17 | 90 |
| 30 | F | 56 | sigmoid | 17 | 95 |
| 31 | M | 78 | sigmoid | 11 | 90 |
| 32 | F | 48 | rectum | 50 | 99 |
| 33 | F | 46 | rectum | 12 | 50 |
| 34 | M | 53 | caecum | 15 | 99 |
| 35 | M | 48 | rectum | 25 | 90 |
| 36 | F | 67 | sigmoid | 20 | 78 |
| 37 | M | 59 | sigmoid | 21 | 85 |
| 38 | F | 57 | sigmoid | 25 | 88 |
| 39 | F | 55 | rectum | 30 | 28 |
| 40 | M | 82 | sigmoid | 10 | 95 |
| 41 | M | 66 | ascending | 18 | 85 |
| 42 | M | 42 | sigmoid | 40 | 93 |
| 43 | F | 67 | rectum | 55 | 95 |
| 44 | M | 59 | sigmoid | 10 | 90 |
| 45 | F | 77 | ascending | 25 | 99 |

| # | sex | age | localization | % of adenocarcinoma |
| --- | --- | --- | --- | --- |
| 1 | M | 69 | transverse | 21 |
| 2 | F | 76 | caecum | 94 |
| 3 | F | 74 | caecum | 97 |
| 4 | M | 63 | ascending | 91 |
| 5 | F | - | rectum | 35 |
| 6 | M | 74 | rectosigmoid | 35 |
| 7 | F | 64 | rectum | 25 |
| 8 | F | 55 | descending | 27 |
| 9 | F | 55 | descending | 27 |
| 10 | M | 78 | rectum | 29 |
| 11 | F | 64 | sigmoid | 92 |
| 12 | M | 50 | sigmoid | 45 |
| 13 | M | 73 | rectum | 48 |
| 14 | F | 66 | descending+sigmoid | 42 |
| 15 | M | 69 | sigmoid | 86 |
| 16 | F | 38 | sigmoid | 50 |
| 17 | M | 63 | rectum | 50 |
| 18 | F | 56 | ascending | 40 |
| 19 | M | 55 | rectum | 80 |
| 20 | F | 62 | descending | 98 |
| 21 | F | 62 | sigmoid | 54 |
| 22 | F | 82 | ascending | 51 |
| 23 | F | 65 | ascending | 39 |
| 24 | M | 54 | descending | 89 |
| 25 | M | 59 | rectum | 61 |
| 26 | F | 76 | ascending | 81 |
| 27 | M | 80 | sigmoid | 15 |
| 28 | F | 78 | ascending | 81 |
| 29 | F | 69 | ascending | 78 |
| 30 | M | 80 | caecum | 77 |
| 31 | F | 61 | sigmoid | 55 |
| 32 | F | 65 | sigmoid | 66 |
| 33 | M | 60 | rectum | 50 |
| 34 | F | 71 | rectosigmoid | 97 |
| 35 | M | 47 | caecum | 80 |
| 36 | M | 58 | transverse | 79 |

| # | sex | age | localization | % normal mucosa |
| --- | --- | --- | --- | --- |
| 1 | M | 77 | caecum | 60 |
| 2 | F | 71 | rectum | 35 |
| 3 | F | 69 | sigmoid | 50 |
| 4 | M | 78 | rectum | 50 |
| 5 | F | 64 | sigmoid | 50 |
| 6 | F | 66 | descending | 80 |
| 7 | M | 69 | sigmoid | 68 |
| 8 | F | 38 | sigmoid | 45 |
| 9 | M | 55 | rectum | 60 |
| 10 | F | 62 | sigmoid | 38 |
| 11 | F | 65 | ascending | 68 |
| 12 | M | 59 | rectum | 85 |
| 13 | F | 61 | sigmoid | 70 |
| 14 | F | 65 | rectum | 45 |
| 15 | M | 74 | ascending | 18 |
| 16 | F | 51 | rectum | 100 |
| 17 | F | 61 | sigmoid | 78 |
| 18 | F | 55 | caecum | Mostly mucosa |
| 19 | F | 62 | ascending | Mostly mucosa |
| 20 | F | 54 | sigmoid | Mostly mucosa |
| 21 | M | 57 | transverse | Mostly mucosa |
| 22 | M | 62 | transverse | Mostly mucosa |
| 23 | F | 50 | sigmoid | Mostly mucosa |
| 24 | F | 50 | rectum | Mostly mucosa |
